# Human representation of multimodal distributions as clusters of samples

**DOI:** 10.1371/journal.pcbi.1007047

**Published:** 2019-05-14

**Authors:** Jingwei Sun, Jian Li, Hang Zhang

**Affiliations:** 1 School of Psychological and Cognitive Sciences and Beijing Key Laboratory of Behavior and Mental Health, Peking University, Beijing, China; 2 PKU-IDG/McGovern Institute for Brain Research, Peking University, Beijing, China; 3 Peking-Tsinghua Center for Life Sciences, Peking University, Beijing, China; Harvard University, UNITED STATES

## Abstract

Behavioral and neuroimaging evidence shows that human decisions are sensitive to the statistical regularities (mean, variance, skewness, etc.) of reward distributions. However, it is unclear what representations human observers form to approximate reward distributions, or probability distributions in general. When the possible values of a probability distribution are numerous, it is cognitively costly and perhaps unrealistic to maintain in mind the probability of each possible value. Here we propose a Clusters of Samples (CoS) representation model: The samples of the to-be-represented distribution are classified into a small number of clusters and only the centroids and relative weights of the clusters are retained for future use. We tested the behavioral relevance of CoS in four experiments. On each trial, human subjects reported the mean and mode of a sequentially presented multimodal distribution of spatial positions or orientations. By varying the global and local features of the distributions, we observed systematic errors in the reported mean and mode. We found that our CoS representation of probability distributions outperformed alternative models in accounting for subjects’ response patterns. The ostensible influence of positive/negative skewness on the over/under estimation of the reported mean, analogous to the “skewness preference” phenomenon in decisions, could be well explained by models based on CoS.

## Author summary

Life is full of uncertainties: An action may yield multiple possible consequences and a percept may imply multiple possible causes. To survive, humans and animals must compensate for the uncertainty in the environment and in their own perceptual and motor systems. However, how humans represent probability distributions to fulfill probabilistic computations for perception and action remains elusive. The number of possible values in a distribution is vast and grows exponentially with the dimension of the distribution. It would be costly, if not impossible, to maintain the probability of each possible value. Here we propose a sparse representation of probability distributions, which can reduce an arbitrary distribution to a small set of coefficients while still keeping important global and local features of the original distribution. Our experiments provide preliminary evidence for the use of such representations in human cognition.

## Introduction

As Horace Barlow wrote, “the brain has to decide upon actions in a competitive, chance-driven world, and to do this well it must know about and exploit the non-random *probabilities* and interdependences of objects and events” [1]. In general, the probabilistic information the cognitive system needs to deal with lies in the form of probability distributions of varying kinds: distributions of sensory stimuli in the natural environment [2–4], distributions of sensorimotor errors for motor actions [5–7], and distributions of rewards and penalties for alternative choices [8, 9]. There is evidence for close-to-optimal probabilistic inference in human perception [10], cognition [11], and motor control [6], suggesting that the cognitive system is capable of coding probability distributions to satisfactory precision. Given that an arbitrary distribution can have myriad possible values and render an exact representation unaffordable, what approximations may be used in human representation of probability distributions?

Three general approaches have been proposed for the internal coding of probability distributions [12]. The first is to represent the event probabilities separately [13, 14]. However, such coding schemes would be practically impossible for continuous probability distributions where the number of potential events is infinite, unless additional discretization procedures are assumed. The second approach is sampling; that is, to represent the values of a set of samples from the underlying distribution [15–17]. Probabilistic inference or decision making, therefore, could be based upon samples harnessed from the underlying distribution [18–21], analogous to Monte Carlo methods. Indeed, there are circumstances where people seem to base their judgment or decision on a few [22] or even one [23, 24] random sample taken from the distribution. Third, any probability distribution, in the form of probability density or probability mass functions, may be approximated by the linear combination of a set of basis functions [25–29], much like the fact that time series can be decomposed into the sum of sine and cosine functions in Fourier analysis. The idea of basis function representations is appealing, because it reduces the whole distribution into a set of coefficients and therefore alleviates the cognitive load subjects would have otherwise undertaken, given that the forms of the basis distributions are known [25]. Recently, Zhang, Daw, and Maloney [30] have provided preliminary behavioral evidence that people might represent their own sensorimotor error distributions with a small number of basis distributions.

A fourth approach, which has not been explicitly proposed but has been the foundation of statistical decision theory, is to encode the moments of probability distributions, such as mean (first), variance (second), skewness (third central moment), and so on [31–33]. Mathematically, the whole sequence of moments of a specific distribution contains all the information of the distribution. There have been a number of empirical studies of economic decision-making where efforts have been made to map brain regions dedicated to the calculation of the first three moments [34–38], with the implicit assumption that different moments might be separately processed by different brain structures.

**Fig 1:**
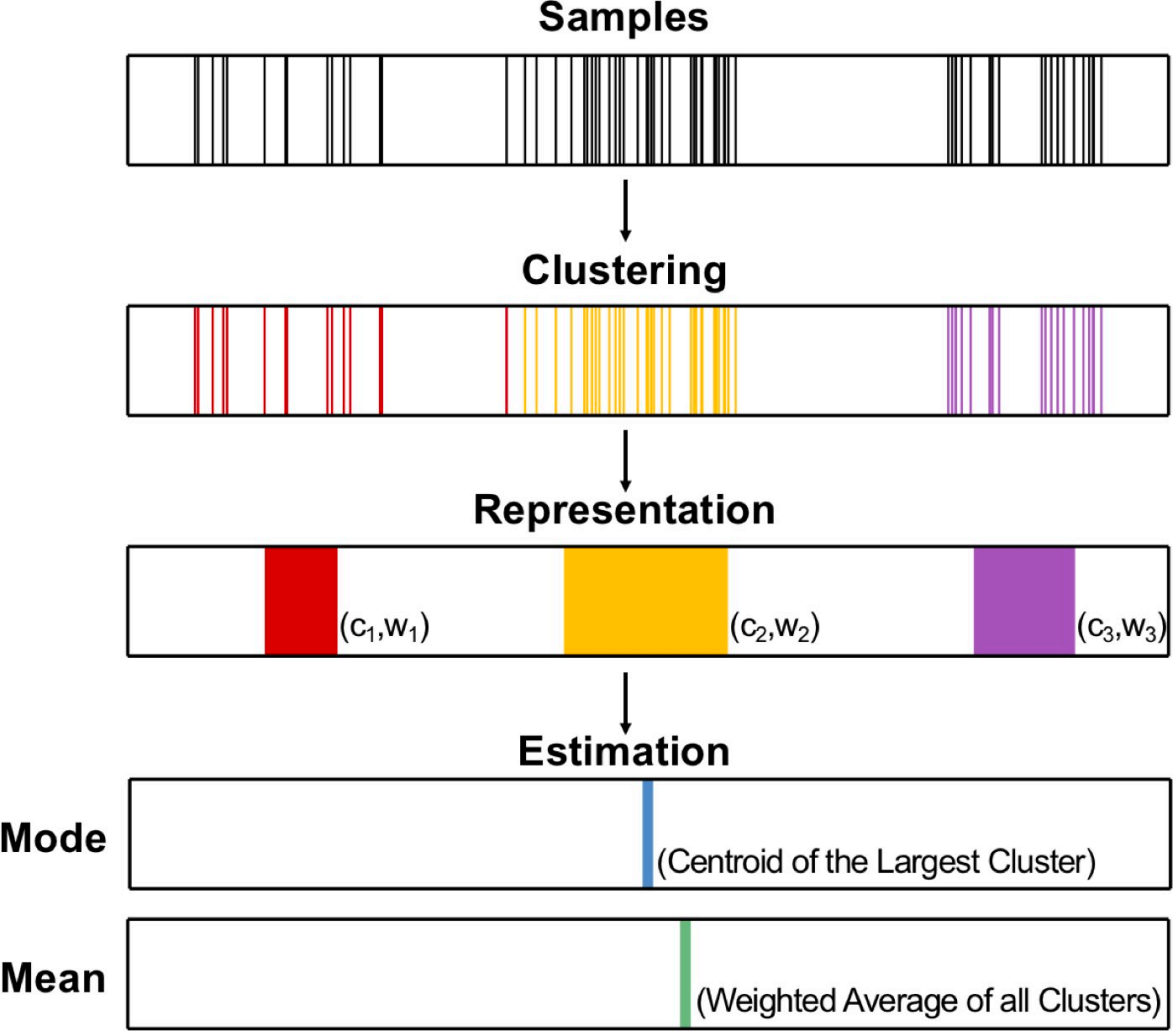
Scheme of the Clusters-of-Samples (CoS) representation. The samples from the empirical distribution are partitioned into a few clusters after a stochastic clustering process and the centroids and relative weights of the clusters are internally maintained for future use (“Representation”). When asked to report the Mode and Mean of the distribution, subjects would report the centroid of the largest cluster as the Mode estimate, and a weighted average of all clusters (whose weights may be subject to additional transformations) as the Mean estimate.

In the present study, we explore the basis function hypothesis where a probability distribution is represented as a set of coefficients of particular basis functions. What is often emphasized in previous theoretical work [25, 28] is the flexibility of this approach. In theory, any probability distribution can be well approximated as long as enough basis distributions are used. Humans in practice, however, may not be able to afford a large number of basis distributions, and the coefficients they extract from the empirical distribution are error-prone. There is increasing evidence that human representations of prior distributions can deviate significantly from the empirical distribution [30, 39] and such deviations prove to be an important source of suboptimality in human probabilistic inference [40]. If humans do not necessarily have a lossless representation of the encoded distribution, a natural question arises: What information is extracted from the empirical distribution? Here we propose the following representation of probability distributions (Fig 1). After a stochastic clustering process, samples from the distribution are classified into a small number of clusters and the centroids and relative weights of the clusters—${\left\{\left(c_{k},w_{k}\right)\right\}}_{k=1}^{K}$—are maintained for future use. We call it Clusters-of-Samples (CoS) representation for which, as in the sampling approach, probabilistic information initially comes from samples and, as in the basis function approach, only a finite set of coefficients needs to be estimated and maintained to approximate the encoded distribution.

An idea akin to our proposal was Shelton et al.’s [41] select-and-sample approach, which assumes that a specific probability distribution is coded by samples but only a small set of pre-selected high-density areas of the distribution may be sampled. Similar to the CoS representation, the select-and-sample approach would reduce an arbitrary distribution to a small number of high-density centers. Shelton et al. [41] proved theoretically that such centers could efficiently approximate multimodal distributions in high-dimensional spaces while retaining the correlations between dimensions. Whereas in our hypothesis, by representing an arbitrary distribution with a set of cluster centers, we are essentially using the clusters as the basis functions to discretize the distribution and obtaining a mixture of Dirac delta functions. The CoS representation can also be extended to the usage of uniform or Gaussian distributions as the bases, with additional coefficients for the spread of the basis distributions.

On the one hand, even with just a small number of clusters, a CoS representation reflects the overall shape of the encoded distribution and is capable of capturing multiple modes if the encoded distribution is multimodal. On the other hand, the CoS representation is prone to information loss. With groups of samples summarized by the cluster centers, information about individual samples in each cluster and thus local details about the distribution are lost. Moreover, the stochasticity in the clustering process may cause variations in the weights as well as in the centroids of the clusters. Due to its characteristic lossy coding, CoS can lead to systematic errors in certain tasks, which would allow us to test CoS against candidate representations that predict no such errors or different patterns of errors. Following the reasoning above, a possible testbed for CoS would be processing multimodal distributions. The main goal of our study is to test how humans cope with the structure of multimodal distributions.

In Marr’s [42] term, our proposal of the CoS representation resides on the computational level, concerning what statistics for a probability distribution are internally maintained. How CoS is implemented algorithmically or biologically, however, is a separate question. In order to make our arguments concrete and testable, we specified certain computational procedures about the stochastic clustering process. In particular, we implemented the stochastic clustering process as a distance-dependent Chinese Restaurant Process (ddCRP) [43]. It has the desirable property that the number of clusters does not have to be specified in advance and is instead determined by a self-adaptive probabilistic process (see Methods). It should also be noted that not all the alternative representations we reviewed earlier are incompatible with CoS on the computational level. For example, a sampling-based representation following the select-and-sample approach [41] may have similar behavioral consequences as CoS.

**Fig 2:**
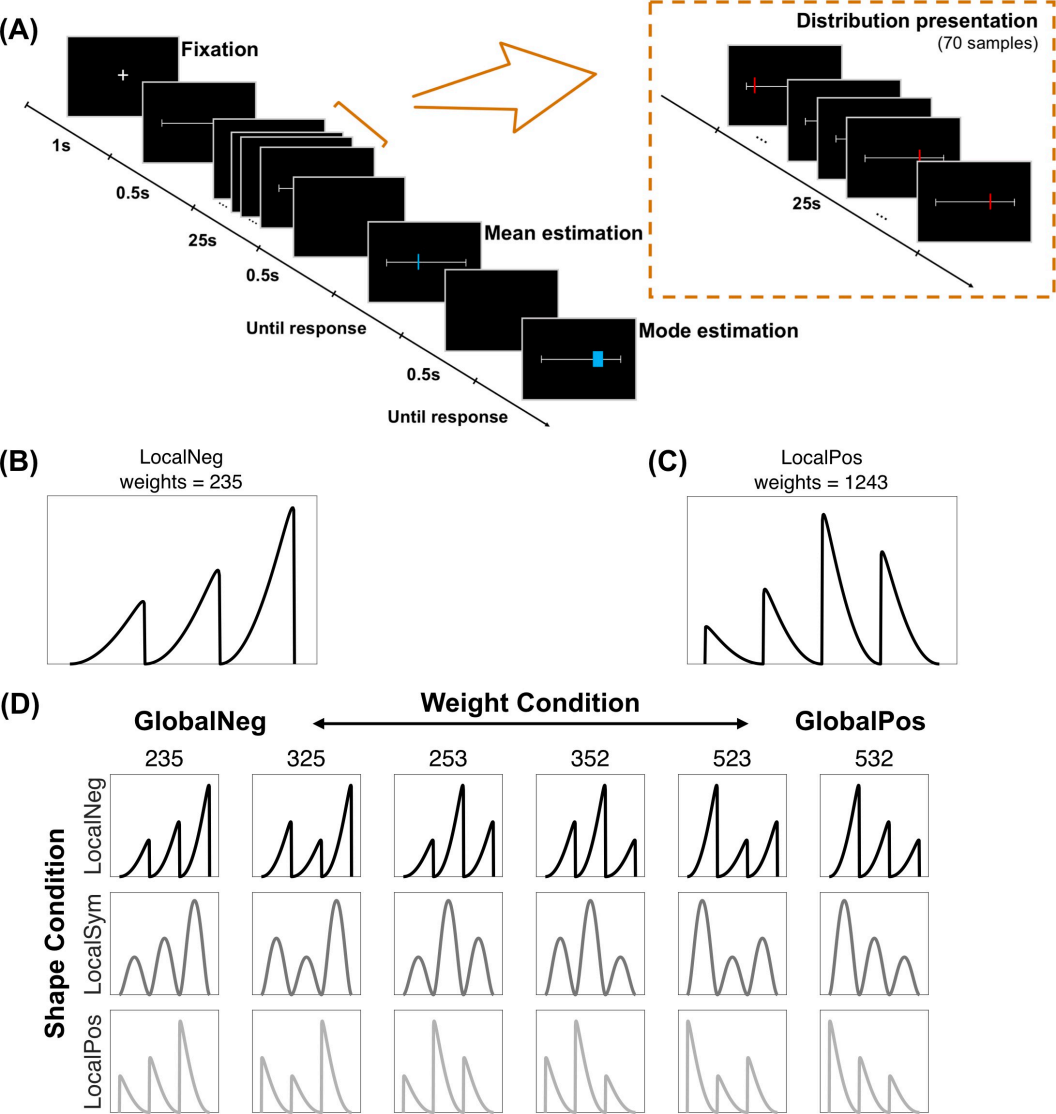
Task and design of the experiments. (A) Time course of one trial. Following a fixation cross, subjects saw 70 red vertical lines sequentially presented on a white horizontal axis. Their task was to move a blue vertical line to indicate their estimate of the Mean (the average horizontal position of the red lines) and then a blue box to indicate the Mode (the area that would catch the largest number of red lines). (B) An example of a 3-beta mix (i.e. mixture of three beta distributions) underlying distribution used in Experiments 1, 3 and S1. This example consists of three negatively skewed beta components (“LocalNeg”), with the weights of the three components from left to right being 0.2, 0.3 and 0.5 (“weights = 235”). (C) An example of a 4-beta mix (i.e. mixture of four beta distributions) underlying distribution used in Experiments 2 and 3. This example consists of four positively skewed beta components (“LocalPos”), with the weights of the four components being 0.1, 0.2, 0.4 and 0.3 (“weights = 1243”). (D) All 3-beta mix underlying distributions used in Experiments 1, 3 and S1. “Pos”, “Sym”, and “Neg” are abbreviations respectively for positive, symmetric, and negative. Local skewness (LocalPos, LocalSym, and LocalNeg) refers to the skewness of the beta components and is controlled by the shape condition. Global skewness (GlobalPos, GlobalSym, and GlobalNeg) refers to the skewness of the whole distribution and is controlled by the weight condition.

In a series of behavioral experiments, we asked human subjects to report the summary statistics of visually presented distributions. On each trial (Fig 2A), 70 vertical lines, whose horizontal coordinates were samples randomly drawn from a specific underlying probability distribution, were briefly and sequentially presented along the middle axis of the computer screen. Subjects’ task was to move a mouse pointer to locate (1) the *Mean* and (2) the *Mode* (location of the highest density) of the observed distribution of spatial positions. Subjects were not required to memorize the spatial positions of individual vertical lines but rather to report the ensemble statistics of spatial positions [44–48] (see [49] for a review of ensemble perception). The Mean and Mode estimation tasks were specifically chosen to test whether subjects’ representations captured both the global features and local details of the empirical distribution. The underlying distributions were generated as the weighted mixtures of multiple evenly-spaced beta-like distributions (Fig 2B–2D). By varying the relative weight of different beta components, for example by assigning more weights to the left or to the right, we were able to manipulate the global distribution to be more positively or more negatively skewed. In contrast, by varying the shape of individual beta components, we modified the local asymmetry of the distribution. Subjects’ Mean and Mode estimates, therefore, afford a unique opportunity to test different representation hypotheses.

Had subjects represented the distribution exactly as it was observed, their Mean and Mode reports would be unbiased estimators about the true values of the Mean and Mode. However, in all four experiments that we tested, systematic deviations between subjects’ estimates and the ground truths of the empirical distributions were detected. We constructed computational models based on the CoS representation and alternative representations and compared different models’ performance in quantitatively predicting subjects’ Mean and Mode estimates. The CoS models outperformed the alternative models for both estimates.

## Results

### Systematic errors in subjects’ estimates

In Experiment 1, the positions of the 70 vertical lines for each trial were randomly drawn from a mixture of three beta-like distributions that adjoined each other. We call this underlying distribution “3-beta mix”, for which both the shape (identical for all the components in the same distribution) and weights of the beta components were varied across trials (Fig 2D). The shape of the beta components could be negatively skewed, symmetric, or positively skewed (see Methods for details). The weights for the three components, from left to right, could be (0.2, 0.3, 0.5), (0.3, 0.2, 0.5), (0.2, 0.5, 0.3), (0.3, 0.5, 0.2), (0.5, 0.2, 0.3), or (0.5, 0.3, 0.2). In what follows, “local skewness” refers to the skewness (shape) of the individual beta components in the 3-beta mix. We refer the skewness of the whole 3-beta mix distribution as “global skewness” here to differentiate from “local skewness”, which relies mainly on the weights of the beta components. Thus the effects of shape and weight conditions correspond to the local and global skewness effects, respectively. In the experiment, each of the 18 combinations of shape and weight conditions was repeated for 9 times, resulting in 162 trials.

“True mode” and “true mean” refer to the statistics computed from the empirical distribution (i.e. the 70 samples, see Methods). All 16 subjects’ Mode and Mean estimates were positively correlated with the true mode and mean (Pearson’s correlation, all *p* < 0.001). Besides, subjects’ Mode estimate was closer to the true mode than to the true Mean (*t*(15) = -7.78, *p* < 0.001), and their Mean estimate was closer to the true Mean than to the true Mode (*t*(15) = -26.36, *p* < 0.001), indicating that subjects did report the two statistics as instructed instead of using the same estimates for the two tasks.

**Fig 3:**
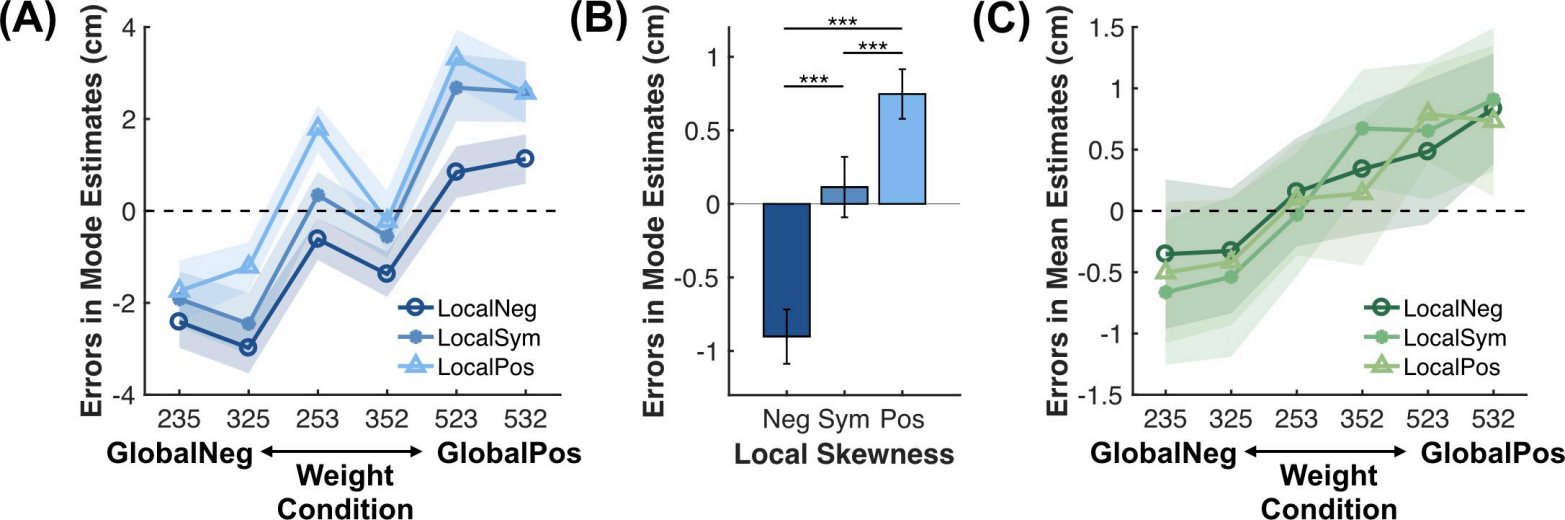
Systematic errors of subjects’ estimates in Experiment 1. (A, C) Subjects’ errors in Mode (A) and Mean (C) estimates varied with the weight condition (abscissa) and the shape condition (different colors). Shaded areas denote 1 SE. See the legend of Fig 2 or the text for the definition of global and local skewness. Different weight conditions are arranged from left to right in increasing global skewness. Note that subjects’ errors in Mode estimates changed non-monotonically with global skewness. See text for intuitions how this non-monotonicity can arise from the CoS representation. (B) The main effect of the shape condition (local skewness) on subjects’ errors in Mode estimates. ***: *p* < 0.001 for Bonferroni corrected post-hoc comparisons. Error bars denote 1 SE.

Meanwhile, the deviations of subjects’ Mode and Mean estimates from the ground truth varied systematically with the shape and weight conditions (Fig 3A and 3C). For subjects’ errors in Mode estimates, a two-way (3 shapes × 6 weights) repeated-measures ANOVA showed significant main effects of shape (*F*(2, 150) = 71.52, *p* < 0.001) and weight conditions (*F*(5, 150) = 199.96, *p* < 0.001) as well as their interaction (*F*(10, 150) = 3.25, *p* = 0.001). Further post-hoc comparisons indicated that the three shape (local skewness) levels differed from each other (all *p* < 0.001, Bonferroni corrected for three comparisons; Fig 3B). A similar ANOVA on subjects’ errors in Mean estimates showed that the main effect of the weight condition (*F*(5, 150) = 39.13, *p* < 0.001) was significant, and no other effects reached the 0.05 significance level.

Three patterns emerged from the behavioral data. First, the Mode was overestimated for positive compared to negative local skewness (Fig 3A and 3B, differences between the three colors). That is, though on average still falling within the beta component where the true mode resided, subjects’ Mode estimate was biased towards the center of the beta component. Second, the Mode was overestimated for positive and underestimated for negative global skewness (Fig 3A, the ascending lines, but note the exceptions at 253 and 352). In other words, the Mode estimate was also biased towards the mean of the empirical distribution.

Third, the Mean was overestimated for positive and underestimated for negative global skewness (Fig 3C, the ascending lines). When choosing among probability distributions of rewards, besides the well-known preference for higher mean (expected gain) and lower variance [50], it has been suggested that people tend to prefer positively skewed over symmetric, and symmetric over negatively skewed distributions. This phenomenon is known as “skewness preference” in economic decisions [51–55], which was considered to be associated with activities in dedicated brain structures devoted to the processing of skewness [34, 56, 57]. Our finding that the reported Mean was positively associated with distribution skewness, other things being equal, echoed previous literature on skewness preference and raised the possibility that skewness preference might be due to the mis-estimation of the mean of skewed reward distributions.

If subjects had an accurate representation of the empirical distribution and computed the required statistics properly, their Mode and Mean estimates would not systematically deviate from the true mode and mean. What representation of probability distributions could best account for the error patterns described above? Based on the different representations introduced earlier, we constructed a variety of models for the estimations of the Mode and Mean, and compared different models’ performance in explaining the data.

### Mode estimates: clusters-of-samples versus alternative models

In the CoS representation of a specific distribution—${\left\{\left(c_{k},w_{k}\right)\right\}}_{k=1}^{K}$, detailed information about the individual samples that constitute each cluster has been lost. As a result, the mode of the distribution cannot be exactly recovered from its CoS representation. In the CoS model for Mode estimates (see Methods), we assume that the subject simply reports the centroid of the cluster that is of the highest weight as the Mode estimate.

**Fig 4:**
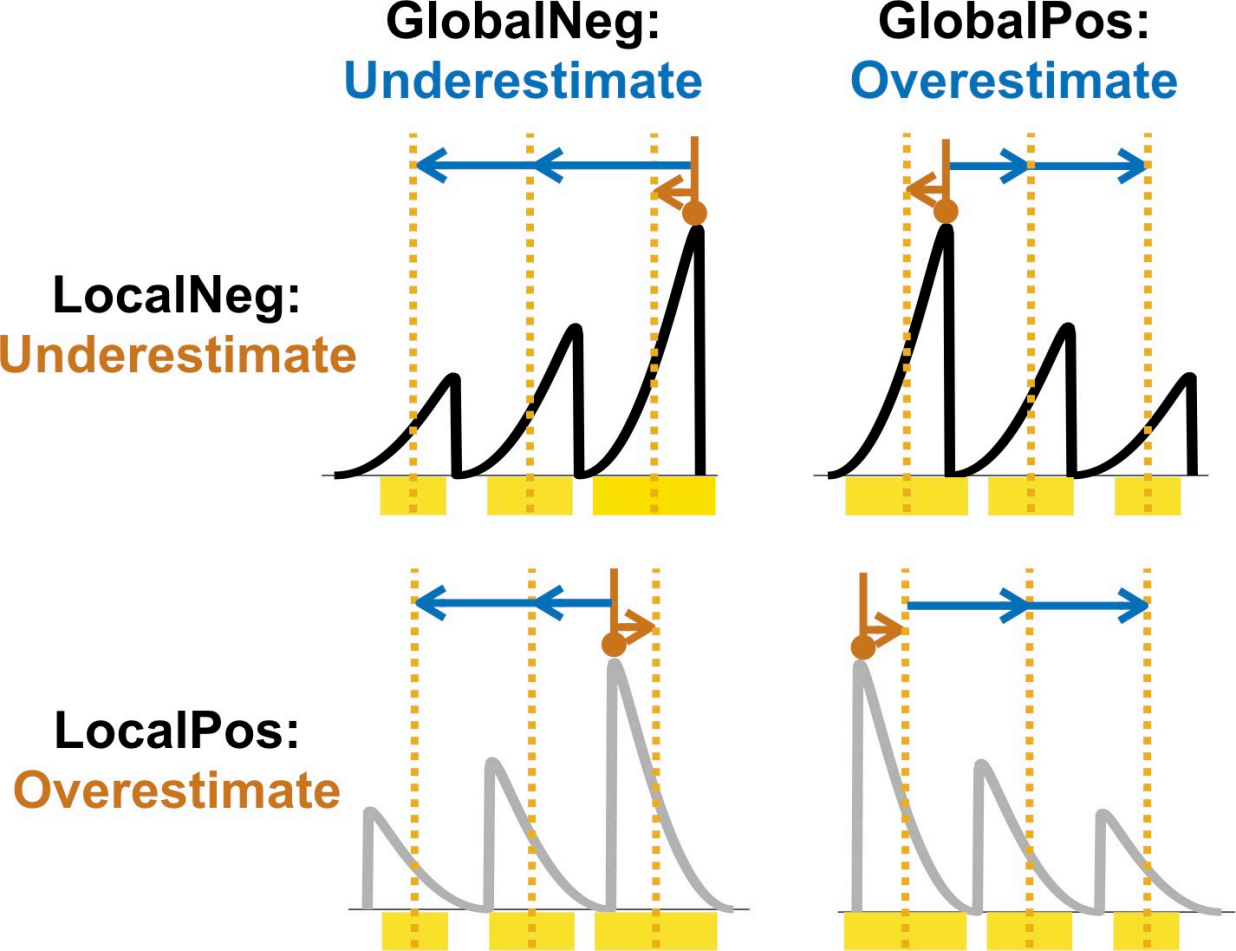
Intuition of how CoS may explain the observed error patterns in Mode estimates. Black or gray curves denote the empirical distribution. Yellow rectangles denote the clusters formed for the CoS representation. Orange dotted lines denote the means of the clusters. The red brown vertical line with circle arrowhead indicates the true Mode. The red brown arrow indicates the influence of local skewness. Given that subjects would report the mean of the largest cluster as the Mode estimate, the mode estimate would be overestimated for locally positively skewed distributions (“LocalPos”) and underestimated for the reverse (“LocalNeg”). Blue arrows indicate the influence of global skewness. Due to the stochasticity of the clustering process, occasionally the largest cluster in the CoS representation does not correspond to the largest beta component, leading to an overestimation of mode for globally positively skewed distributions (“GlobalPos”) and underestimation in the reverse case (“GlobalNeg”).

**Fig 5:**
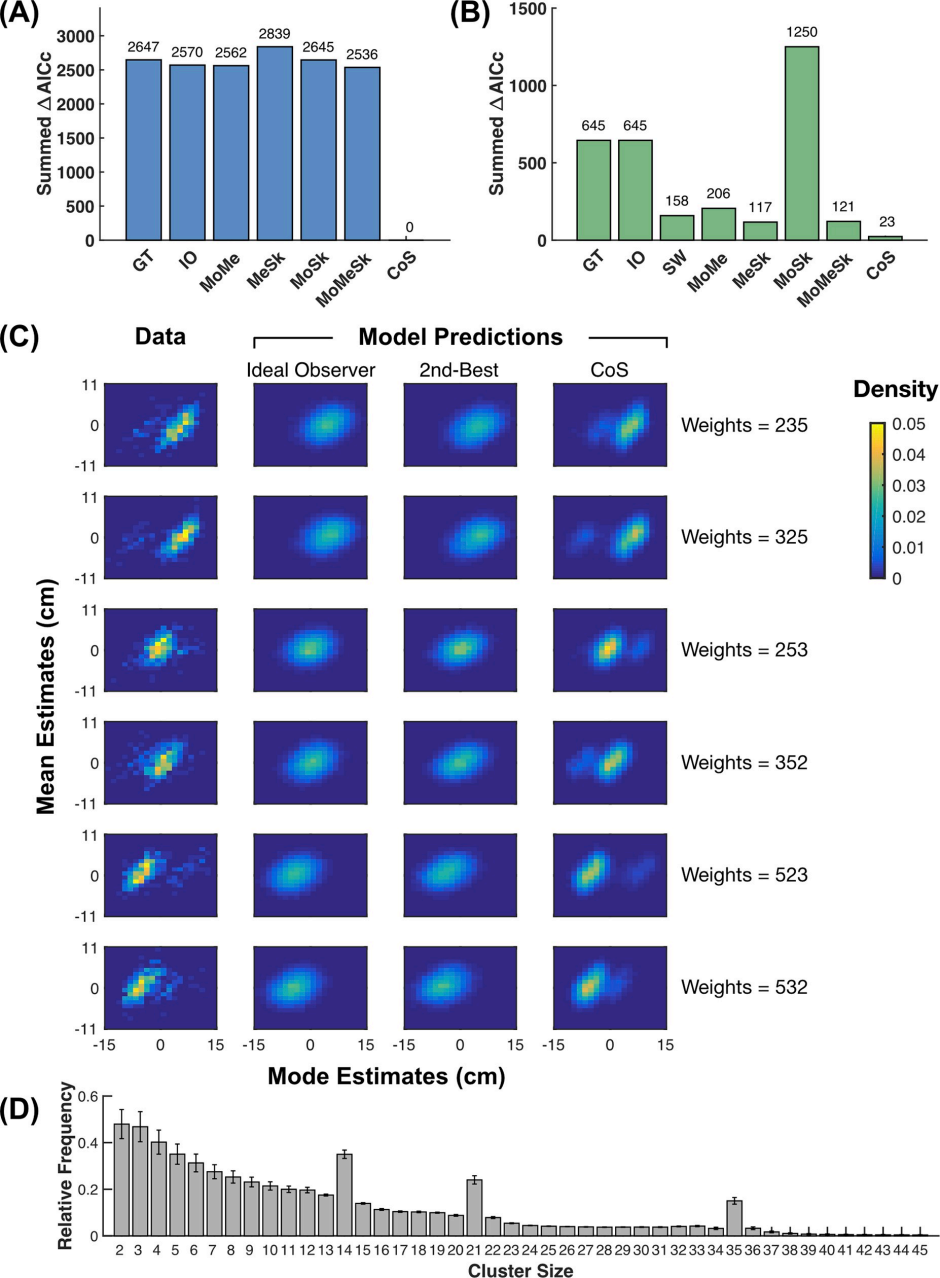
Modeling results of Experiment 1. (A, B) Summed ΔAICc (lower is better) for Mode estimates (A) and Mean estimates (B). GT: ground-truth model; IO: ideal observer model; MoMe: mode+mean model; MeSk: mean+skewness model; MoSk: mode+skewness model; MoMeSk: mode+mean+skewness model; CoS: clusters-of-samples model; SW: subjective weighting model. (C) Joint distributions of Mode and Mean estimates collapsed across subjects for data, the ideal observer models, the moment-based second-best models (mode+mean+skewness for Mode and mean+skewness for Mean) and the CoS models. (D) Relative frequency of different cluster sizes (number of samples per cluster) estimated for subjects’ CoS representations. For each subject, the relative frequency of each cluster size was averaged across trials and possible CoS representations on each trial. The statistics were then averaged across subjects. Error bars denote 1 SE. Overall, the clustering process had a tendency to generate clusters of small sizes. The frequencies for cluster sizes of 14, 21, and 35—which correspond to the three relative weights used in 3-beta mix—were much higher than those of their neighbors, indicating that the clustering process could partly recover the multimodal structure of the empirical distribution.

Intuitively, the CoS model can produce the observed two error patterns of Mode estimates (Fig 4): On the one hand, because the CoS representation is ignorant of the individual samples of each cluster and only identifies their means, the CoS model would predict that the mean—instead of the mode—of the largest beta component mainly drives subjects’ Mode estimates. On the other hand, due to the stochasticity of the clustering process, the largest cluster in the CoS representation occasionally does not correspond to the largest beta component. Thus, on average, the Mode estimate would deviate from the mean of the largest beta component towards the mean of the whole distribution. In addition, the occasional mismatch of the largest cluster in the CoS representation with the largest beta component in the underlying distribution gives rise to two more specific predictions for Mode estimates, both of which are supported by our data. First, it predicts that subjects’ Mode estimates across trials would be multimodally distributed, with the major peak centered at the largest beta component and a minor peak at the second largest beta component (see Fig 5C for our further specifications of the data and model predictions). Second, it can naturally predict the observed non-monotonic increase of errors with global skewness (Fig 3A): Given that the 253 weight condition has its second largest beta component on the right of its largest beta component and the 352 condition has the reverse arrangement, the former is likely to incur a more positive error than the latter, though the former is associated with negative global skewness and the latter with positive global skewness.

We considered several alternative models for Mode estimates. One model is the ground-truth model, where the Mode estimate is assumed to be the same as the true mode. Apparently the ground-truth model cannot explain any systematic biases from the true mode and therefore only serves as a baseline for the other models.

The second model is a Bayesian ideal observer model (see Methods), in the consideration that even an ideal observer may not be able to build an accurate representation of the empirical distribution from the available samples and thus may show certain biases. Following Orhan and Jacobs’ [58] modeling of working memory, we used a Dirichlet Process Mixture Model (DPMM) as the generative model assumed by the ideal observer. That is, the ideal observer assumes a “bumpy” world: Each observed sample descends from a specific cluster and its value is generated from the Gaussian distribution associated with the cluster. The number of clusters and the number of samples in each cluster are assumed to follow a Dirichlet random process. DPMMs are commonly used to model cognitive processes [58–60], which have the desirable property that the number of clusters need not to be pre-specified but can be estimated from the data. By estimating the parameters of such a generative model from the observed samples, the ideal observer can approximate the empirical distribution with a Gaussian mixture distribution, whose mode would be reported as the Mode estimate. We found that the Gaussian mixture distribution obtained by the ideal observer closely matches the empirical distribution, even for beta mixtures that have skewed beta components (S4 Fig). That is, the behavior responses of the Bayesian ideal observer model would be almost equivalent to those of the ground-truth model and not show systematic errors.

The other alternative models are based on a moment-based representation of probability distributions, all of which can qualitatively reproduce, at least part of, the observed error patterns in Mode estimates. The moment representation does not necessarily suggest any biases: In theory, the mode of a distribution can be recovered from the set of moments of the distribution. However, if subjects only represented the first a few moments and used them to estimate the mode, or if subjects had an unbiased internal estimate of the true mode but their responses were biased by certain task-irrelevant moments, their responses might show systematic errors. To test these possibilities, we constructed a series of moment representation models where the Mode estimate is a weighted average of the true mode and moments of the distribution plus random noises. In particular, the models were mean+skewness, mode+mean, mode+skewness, and mode+mean+skewness. We did not include variance as a predictor in these models since all the distributions were generated with equal variance.

**Fig 6:**
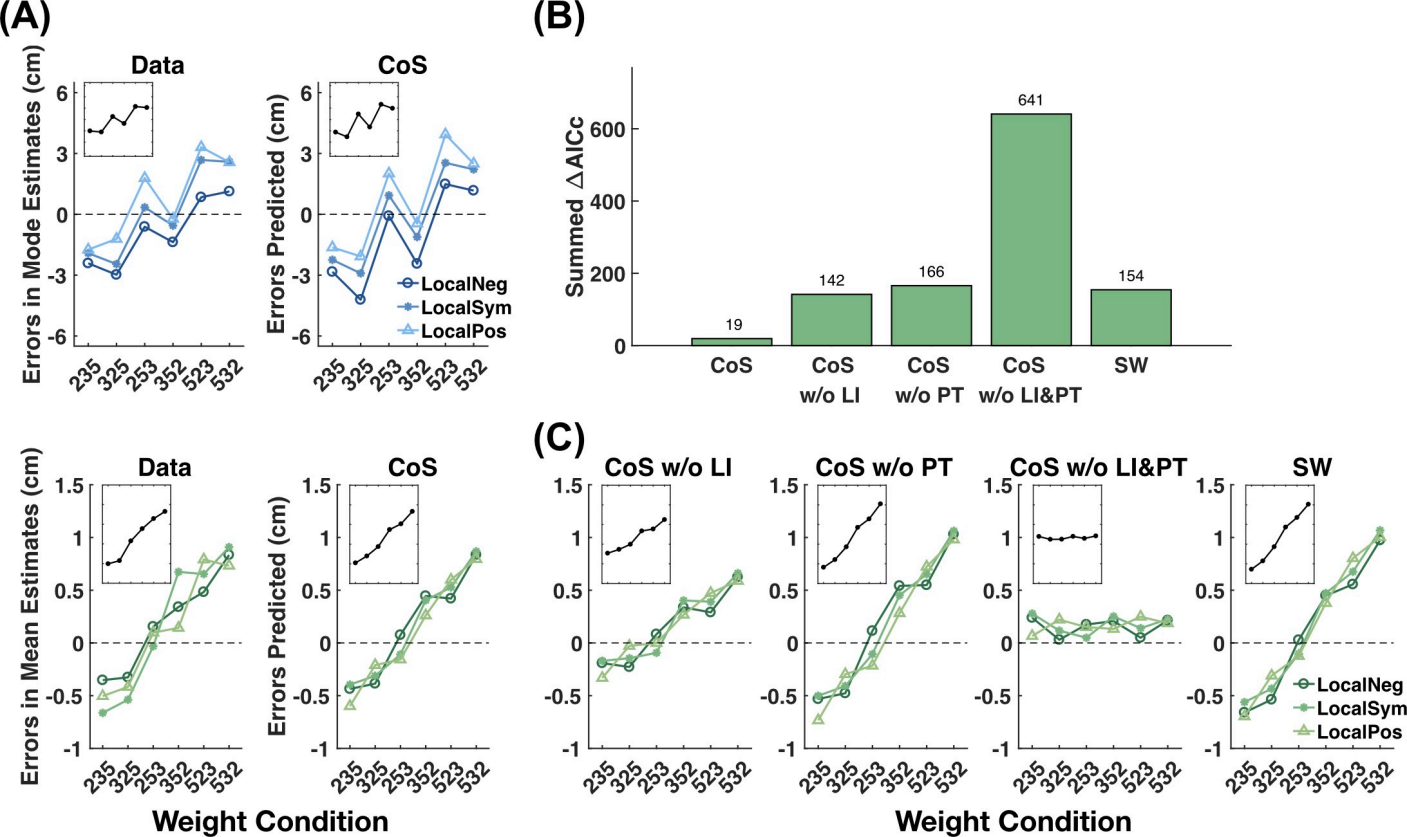
Model predictions and model lesion analysis for Experiment 1. (A) Subjects’ errors in Mode and Mean estimates (left panels, the same as Fig 3A & 3C) versus the predictions of the CoS models (right panels). The CoS models could well predict subjects’ errors. Insets refer to the mean across the three local skewness conditions. (B) Summed ΔAICc of the CoS model for Mean estimates and its lesioned models. The lesioned models included models without Lateral Inhibition (“CoS w/o LI”), without Power Transformation (“CoS w/o PT”), without both the components (“CoS w/o LI&PT”), and without clustering (“SW”, which is the subjective weighting model in Fig 5B). The unlesioned CoS model outperformed the lesioned models, implying that power transformation, lateral inhibition and the clustering process were all necessary for the CoS model to explain subjects’ Mean estimates. (C) Predictions of the four lesioned CoS models, which deviated from the data quantitatively or qualitatively. Given the models’ considerable differences in ΔAICc, their differences in the prediction plots might look counterintuitively small, which is partly due to the fact that these predictions are only about the mean trend of data whereas different models can also differ in their noise distributions. See text for the intuitions for these results.

All models share a common assumption that the final Mode response undergoes an additional linear transformation and contains a Gaussian random noise, due to the imperfect mapping from perception to motor response. We fit each model to each subject’s Mode estimates using maximum likelihood estimates and computed the Akaike information criterion corrected for small sample-size (AICc) [61, 62] as the metric of goodness-of-fit for model comparison. The ΔAICc of a specific model for a specific subject is defined as the difference between the AICc of the model and the lowest AICc for the subject. According to the summed ΔAICc across subjects (Fig 5A), the CoS model was the best predictor of Mode estimates. A group-level Bayesian model selection [63, 64] showed that the protected exceedance probability of the CoS model, i.e. the probability for the CoS model to outperform all the other models' predictions of Mode estimates, was close to 100%. The CoS model well predicted the systematic errors in subjects’ Mode estimates, including the non-monotonic increase of errors with global skewness and the effect of local skewness (Fig 6A).

### Mean estimates: clusters-of-samples versus alternative models

On each trial, subjects reported both the mean and mode of the empirical distribution. It is reasonable to assume that the two estimates are based on a shared CoS representation. The exact CoS representation for a specific trial is not deterministic because the stochastic clustering process may end up with different clustering results and thus different CoS representations on different runs. However, subjects’ Mode estimates had allowed us to infer a probability distribution over different CoS representations for each trial (see Methods) and we used this information to model subjects’ Mean estimates. Given a specific CoS representation ${\left\{\left(c_{k},w_{k}\right)\right\}}_{k=1}^{K}$, we assume that the Mean estimate is a weighted average of all *c*_*k*_, where the subjective weight for *c*_*k*_ is a transformation of *w*_*k*_ that reflects probability distortion [65] and lateral inhibition [66, 67].

We hypothesize that the systematic errors in subjects’ Mean estimates arise as a consequence of CoS representations followed by further transformations upon CoS. The CoS representation itself would not lead to any systematic errors in Mean estimates, because the relative weight of each sample in computing the mean is faithfully transferred to the cluster it is assigned to and thus effectively not altered by the clustering process. Probability distortion and lateral inhibition, as we specify later in a model lesion analysis, would cause overweighting or underweighting of different parts of the distribution and thus biases in Mean estimates. To exclude the possibility that the observed biases are solely induced by the additional transformations, we constructed an alternative model for Mean estimates with similar transformations but no clustering—the subjective weighting model (see Methods), and compared its performance with that of the CoS model (Fig 5B & Fig 6B and 6C).

A Bayesian ideal observer model and several moment representation models for Mean estimates were defined in a similar way as their counterparts for Mode estimates (see Methods). Parallel to the models for Mode estimates, all models for Mean estimates include a linear transformation and Gaussian noise. The model fitting and comparison procedures were the same as those of Mode estimates.

The CoS model outperformed the other models for Mean estimates in the summed ΔAICc. According to a group-level Bayesian model selection, it had a 99.5% probability to excel all the other models (Fig 5B). Note that the mean+skewness model—Mean estimate as a weighted average of the mean and skewness of the distribution—was among the models that were inferior to the CoS model, though it seems to provide a straightforward explanation for the “skewness preference” (Fig 3C). Similarly, though assuming a lateral inhibition between samples can cause samples underweighted in dense areas and overweighted in sparse areas and thus explain the “skewness preference” (S5 Fig), the subjective weighting model without clustering fit worse to the data than the CoS model did.

The CoS model for Mean estimates (but not that for Mode estimates) includes additional transformations. In a model lesion analysis, we tested further how the additional transformations as well as the clustering process of the CoS model contributed to its performance in Mean estimates. When lateral inhibition, power transformation, both the transformations, or clustering were removed from the CoS model (the CoS model w/o clustering is equivalent to the subjective weighting model in Fig 5B), the resulting model fit worse to subjects’ Mean estimates than the CoS model did (Fig 6B, also see S7 Fig for similar results of further experiments). Compared to its lesioned versions, CoS had a smaller summed ΔAICc and a protected exceedance probability of 95.6%. The CoS model well predicted subjects’ errors in Mean estimates (Fig 6A), whereas the lesioned models failed quantitatively or qualitatively (Fig 6C). The failure of the CoS model without both transformations (i.e. the CoS w/o both model) is straightforward since the CoS representation alone does not introduce biases into Mean estimates, as we mentioned earlier. The intuitions for the other two lesioned models are as follows. When there is no lateral inhibition (i.e. the CoS w/o LI model), power transformation leads to a re-distribution of cluster weights that depends only on the values of the weights but not on how the clustered are aligned. As we elaborate later, the major clusters in subjects’ CoS representations closely followed the beta components in the empirical distribution. Therefore, the weight re-distributions would be similar for the 235 and 325 weight conditions (weights moving from the rightmost cluster to the left two clusters) and for the 523 and 532 weight conditions (weights moving from the leftmost cluster to the right two clusters). As the result, the CoS w/o LI model predicted an *S*-shaped error pattern (left panel of Fig 6C). In contrast, when there is lateral inhibition but no power transformation (i.e. the CoS w/o PT model), the re-distribution of cluster weights depends on the alignment of the clusters, for which 235 and 325, or 523 and 532 would be rather different. Consequently the CoS w/o PT model predicted a close-to-linear error pattern (central panel of Fig 6C). The error patterns predicted by the CoS model as well as that of the data lay between those of the two lesioned models.

To see whether subjects really, as we assumed, reported their Mode and Mean estimates based on a common CoS representation, we constructed an additional lesioned CoS model for Mean estimates that does not use the representational information inferred from the Mode estimate on the same trial. If two distinct CoS representations had been used for Mode and Mean estimates, the CoS representation inferred from the Mode estimate would be non-informative for predicting the Mean estimate and the lesioned model would perform equally well as the original model. Instead, we found that this lesioned model was inferior to the original CoS model in fitting subjects’ Mean estimates (S6 Fig), thus providing evidence for a shared CoS representation across the two estimation tasks.

### Multimodality and statistics of the clusters

Though more than one model can qualitatively predict the error patterns of Mode and Mean estimates in Fig 3, the CoS models outperformed the alternative models in predicting the full distributions of Mode and Mean estimates. Fig 5C shows the joint distributions of Mode and Mean estimates, collapsed across subjects and separately for the six weight conditions, compared between data and model predictions. In most of the data plots, the joint distributions appeared to be multimodal: We can see at least two separate peaks, one dominant and the other minor (see S9 Fig for statistical evidence). The major peak for each weight condition corresponds to the beta component of the largest weight, while the minor peak corresponds to the second-largest beta component. The CoS models predicted such multimodality, while the moment representation models predicted only unimodal distributions. The CoS representation could lead to the observed multimodality in Mode estimates because of the stochasticity inherent in its clustering process so that the same empirical distribution may be parsed into different partitions in different runs.

To illustrate how the clusters in subjects’ CoS representations can capture the statistics of the empirical distribution, we plot the relative frequencies of different cluster sizes (i.e. number of samples per cluster, see Fig 5D), which were averaged across trials and possible CoS representations on each trial. The frequencies for 14, 21, and 35 samples per cluster—which correspond to the relative weights of 0.2, 0.3, and 0.5 that were used in our stimuli—were much higher than those of their neighbors, indicating that the clustering process could partly recover the multimodal structure of the empirical distribution.

To test whether our findings in Experiment 1 can generalize to decision contexts other than spatial position judgments, we performed Experiment S1 (see Methods and S8 Fig), which used the same design as Experiment 1 but replaced spatial positions with orientations—another widely used stimuli in ensemble perception [49]. The results of Experiment S1 (S3 Fig) replicated the major findings of Experiment 1, including the three error patterns in Mode and Mean estimates, the superiority of the CoS models over the alternative models, and the multimodal distributional structure captured in subjects’ CoS representations. We also noted a slight difference between the results of the orientation and the spatial position experiments: the CoS model for Mean estimates in Experiment S1 had a considerably lower protected exceedance probability compared to its counterpart in Experiment 1.

In Experiment 1, we found that subjects might be sensitive to the multimodality of the distribution and formed clusters accordingly. To further test this hypothesis, we conducted two new experiments, where the task structures were similar to Experiment 1 but the number of modes in the underlying distributions were varied. In Experiment 2, we increased the number of modes to four. In Experiment 3, we had separate mini-blocks where the numbers of modes were different (three or four).

### Experiment 2: 4-beta mix

Sixteen new subjects took part in Experiment 2, where the underlying distribution in each trial was a mixture of four beta-like distributions, abbreviated as 4-beta mix (Fig 2C). As in Experiment 1, we found systematic deviations of the Mode and Mean estimates from the true mode and mean (S1 Fig): Both errors were significantly influenced by the weight condition, *F*(23, 644) = 53.27, *p* < 0.001, and *F*(23, 644) = 13.58, *p* < 0.001, according to repeated-measures ANOVAs. For Mode estimates, the main effect of the shape condition (*F*(2, 644) = 4.83, *p* = 0.008) and its interaction with the weight condition (*F*(46, 644) = 2.87, *p* < 0.001) were also significant. Post-hoc comparisons (Bonferroni corrected for three comparisons) showed that the errors for locally-negatively-skewed distributions were significantly more negative than those of the locally-symmetric (*t*(14) = -3.684, *p* = 0.008) and locally-positively-skewed distributions (*t*(14) = -4.481, *p* < 0.001), while the difference between the latter two conditions was insignificant (*t*(14) = 0.011, *p* = 1.000). There were no other significant effects for Mean estimates.

Again, the Bayesian ideal observer model was able to perfectly recover the underlying 4-beta mix distribution (S4B Fig), which could not explain the systematic errors in Experiment 2. Among the models developed for Experiment 1, the CoS models still performed the best in predicting subjects’ Mode and Mean estimates in Experiment 2, according to summed ΔAICc (S1 Fig). The probabilities for the CoS models to excel the alternative models (i.e. protected exceedance probability) were 100.0% and 93.6%, respectively for the Mode and Mean estimates.

Similar to Experiment 1, subjects’ CoS representations could capture the multimodal structure of the empirical distribution: Clusters of 7, 14, 21, and 28 samples, which correspond to the relative weights of 4-beta mix, stood out from the histogram (S1F Fig).

### Experiment 3: interleaved 3-beta and 4-beta mix

In Experiment 3, we tested whether our CoS model is flexible enough to adapt to a dynamic environment where 3-beta mix and 4-beta mix distributions were presented in alternating mini-blocks of 10–14 trials. As in Experiments 1 and 2, all subjects’ Mode and Mean estimates were significantly correlated with the true mode and mean (Pearson’s correlation, all *p* < 0.001) but had systematic errors (S2 Fig), with the error patterns of the 3-beta and 4-beta trials respectively resembled those of Experiments 1 and 2. We performed 2 (shape conditions) × 2 (weight conditions) repeated-measures ANOVAs separately for 3-beta and 4-beta trials. For 3-beta trials, the main effects of the shape condition (*F*(2, 150) = 9.43, *p* < 0.001) and the weight condition (*F*(5, 150) = 82.73, *p* < 0.001) were significant for Mode estimates, while only the main effect of the weight condition was significant for Mean estimates (*F*(5, 150) = 22.78, *p* < 0.001). For 4-beta trials, the main effects of the shape condition (*F*(2, 690) = 3.6, *p* = 0.028) and the weight condition (*F*(23, 690) = 32.48, *p* < 0.001), and their interaction (*F*(46, 690) = 1.95, *p* < 0.001) were significant for Mode estimates, while only the main effect of the weight condition (*F*(23, 690) = 4.36, *p* < 0.001) was significant for Mean estimates.

The results of model comparisons replicated those of Experiments 1 and 2: The CoS models fit best to both the Mode and Mean estimates, according to summed ΔAICc (S2 Fig). On the group level, the probabilities for the CoS models to outperform the alternative models approached 100% and were 99.9%, respectively for the Mode and Mean estimates. The frequency statistics of cluster sizes of the 3-beta and 4-beta trials mimicked those of Experiments 1 and 2.

## Discussion

We proposed clusters-of-samples (CoS) as an approximate representation of probability distributions and tested it against a variety of alternatives in four behavioral experiments. Human subjects were required to report the Mean and Mode of various multimodal probability distributions and systematic errors were observed in their estimates. We found that models based on the CoS representation accounted for subjects’ error patterns better than alternative models that implement more straightforward explanations based on moment representations.

### The moment representation for probability distributions

The fact that probability distributions can be represented by their central moments—mean, variance, skewness, etc.—has gained increasing popularity in cognitive and decision neuroscience research. Though it was not among the theoretical possibilities formally proposed [12], the moment representation was implicitly assumed in the decision making studies that attempted to separate the brain regions for mean, variance, and skewness [34, 37, 38]. These studies tested subjects’ preferences for different reward distributions to see how the choice-related neural activities may vary with specific moments of the reward distribution. However, concerning the coding of the moments higher than the mean (i.e. variance and skewness), the brain regions identified by recent neuroimaging studies were rather inconsistent: Variance representation was associated with anterior cingulate cortex [34] or ventral striatum and anterior insula [38]; skewness was associated with dorsal insula [34], ventral striatum [38], or anterior insula and dorsomedial prefrontal cortex [37].

These conflicting findings raised the possibility that variance and skewness might not be the variables actually encoded in the brain. Instead, their influences on human decisions may come through another set of more basic variables humans adopt to appraise uncertainty. Indeed, we found that the moment-based models were inferior to the CoS models in fitting both the Mode and Mean estimates, even though the coding of task-irrelevant skewness seems to be a natural explanation for the increase of the Mode and Mean estimates with the skewness of the distribution. An obvious failure of the moment-based models was their inability to capture the multimodality of subjects’ responses.

### CoS and skewness preference

The phenomenon of skewness preference—positively skewed reward distributions are favored over symmetric, and symmetric over negatively skewed reward distributions—have been widely reported in animal studies [57] and in economics and finance [34, 53, 54, 56]. Recent studies started to look into the neural basis of skewness preference, in an attempt to identify the brain regions dedicated to the processing of skewness [34, 37, 38]. However, as discussed earlier, identification of brain regions dedicated in skewness processing is still actively debated and the proposition of skewness (or variance) preference was inconsistent with emerging behavioral results. For example, Strait and Hayden [35] reported a non-monotonic preference ranking of reward distributions in monkeys where weakly negatively skewed reward distributions were less preferable to weakly positively skewed reward distributions and the latter were further less preferable to strongly negatively skewed reward distributions.

Alternative explanations other than explicit representation of skewness have been proposed for skewness preference. For example, Genest, Stauffer, and Schultz [57] measured the utility function for individual monkeys and found that the skewness preference in monkeys’ choices can be accounted for by utility maximization, given that the monkeys’ utility functions are convex. This explanation, however, might have trouble generalizing to the skewness preference observed in human choices [34, 56], because humans’ utility functions are typically concave [68, 69]) and would predict the opposite behavior. Indeed, a recent empirical study of decision under risk in humans [70] reported the coexistence of skewness preference with concave utility functions.

The findings of the present study suggest a new possibility: skewness preference can be the epi-phenomenon of mis-estimating the mean—the expected value of the distribution. We found that subjects overestimated the mean of positively skewed distributions and underestimated the mean of negatively skewed distributions. This would appear to be skewness preference if subjects had been asked to choose between two distributions to maximize the expected value of their choice. In accordance with our conjecture, one recent neuroimaging study showed that higher skewness of reward distributions would lead to stronger activation in ventral striatum [38], a brain region involved in the representation of expected value [71, 72].

Since no preference judgment was involved in our task, the patterned errors we found in subjects’ Mean estimates cannot be an effect of non-linear utility functions. Instead, we showed that an approximate representation of probability distributions along with subsequent distortions of probabilities, as implemented in the CoS model for Mean estimates, may give rise to the “skewness preference”.

### CoS as a simplified representation of probability distributions

Representing probability distributions in the real world, which are often high-dimensional and multimodal, confronts human cognition with potential problems such as the curse of dimensionality [28, 73–76]. The CoS representation proposed here provides a simplified representation of probability distributions by reducing an arbitrary distribution to a few pairs of summary statistics ${\left\{\left(c_{k},w_{k}\right)\right\}}_{k=1}^{K}$. Though coming at the cost of information loss, such simplification is likely to alleviate the mnemonic and computational load in probabilistic inference and decision making.

As a basis-function representation [25, 29, 30], CoS adds to extant sparse-coding approaches to simplifying the representation and computation of probabilistic information [75, 77, 78]. Zhang, Daw, and Maloney [30] investigated subjects’ internal representation of their own visuo-motor error distributions in a motor choice task and found empirical evidence for the basis-function representation. Though the objective distribution was unimodal and close to Gaussian, they found that subjects’ internal representation was multimodal and, among a variety of distribution families, was best fit by the mixture of a small number of non-overlapping basis functions. What remains unknown, however, is how subjects’ internal representation arises from the empirical distribution. Here, with the CoS representation, we attempted to bridge the gap between an empirical distribution and its internal representation through a stochastic clustering process.

We showed that a Bayesian ideal observer that is unaware of the generative process of the distribution stimuli used in our experiments can form an accurate representation of the empirical distribution based on Gaussian mixtures (S4 Fig). As a result, the ideal observer model failed to account for the systematic errors in subjects’ Mode and Mean estimations, but rather performed close to the ground-truth model (Fig 5A and 5B). The CoS model differs from the ideal observer model in several aspects. First, CoS does not involve Bayesian inference and the stochasticity inherent in clustering may not be eliminated even with a large number of samples observed. Second, by only keeping the cluster centers, CoS loses higher-order information about each cluster such as the local skewness. As we reasoned earlier, these characteristics allow CoS to explain the specific error patterns in subjects’ Mode estimates. The combination of CoS and additional transformations but not the transformations alone can also explain subjects’ Mean estimates. By comparing CoS with the ideal observer model in predicting human data, we have obtained evidence for the two key assumptions of the CoS representation: stochastic clustering and the loss of local information.

Our results raise the possibility that inferring the generative process from the observed samples might not occur for a complicated generative process. Even when the form of the generative model is simple and known, a recent study [79] found that people tend not to estimate the generative model. On each trial of their task, subjects saw an array of four dots distributed around a vertical line and were explicitly informed that the horizontal coordinates of the dots were generated from a Gaussian distribution centered at the line. Subjects were required to locate the range that would include 65% of the distribution. Despite comprehensive feedbacks after each trial, subjects’ behavioral patterns systematically deviated from those predicted by a Gaussian internal model. Instead, subjects’ internal model was well approximated by a kernel density estimation based on the four samples, which was consequently multimodal. Though there is doubt whether the kernel density representation holds for distributions presented by more than four samples (see [30] for opposing evidence), the results of [79] as well as our own study suggest that humans may not function as Bayesian ideal observers in probability density estimation.

The CoS representation we proposed also echoes the spontaneous clustering process theorized in the memory literature, for both working memory [58, 80] and long-term memory [81, 82]. According to Orhan and Jacobs [58], people assume the world is “bumpy” and try to infer the clusters from which individual items have been generated. Though their task was to memorize individual items, subjects’ biases during retrieval suggest that the inferred clusters were also maintained and used to compensate for perceptual and mnemonic noises. What we considered here is a different situation, where the task was not to memorize individual items but to report the summary statistics of a distribution. That is, subjects were nudged to extract a representation of the distribution from the samples. While an ideal observer can almost perfectly recover the underlying distribution used in our experiments (S4 Fig), human behavioral data suggested that the local features of the distribution were lost.

### Limitations and future directions

For Gaussian or any symmetric unimodal distributions, mean, median, and mode are all the same. In such cases, it would be theoretically difficult to tell apart different hypotheses about human representations of probability distributions. That is why highly skewed [40, 83, 84] or multi-modal [6, 40, 85] distributions have been used to investigate how people represent probability distributions. Indeed, the specific one-dimensional multimodal distributions we used in the present study revealed diagnostic error patterns, which provided preliminary evidence in support of the CoS representation. But it is still an empirical question whether CoS well describes human representations of probability distributions that are beyond multimodal distributions.

In theory, the CoS representation is applicable to distributions of a higher dimension. It can also be extended to accommodate modulations from top-down cognitive processes by assuming that the parameters controlling the clustering process may be modulated by prior knowledge. Further empirical tests in a broader range of tasks would be required to establish the CoS representation as a general heuristic in representing probability distributions.

## Methods

### Ethics statement

The experiments had been approved by the Institutional Review Board of School of Psychological and Cognitive Sciences at Peking University. Informed consent was given by all subjects prior to the experiments.

### Experiments

Sixty-four paid subjects (19–25 years old, 36 females) participated in the four experiments, with 16 subjects for each experiment. All of them were naïve to the goal of our study. Subjects whose Mode or Mean estimates failed to show significant correlations with the corresponding true statistics were excluded from further data analysis. Only one subject from Experiment 2 was excluded.

Stimuli were presented on black backgrounds on a 52.0×32.5-cm computer screen (1920×1200 px, refresh rate 60 Hz) controlled by Matlab and Psychophysics Toolbox [86–88] and were viewed by subjects from a distance of approximately 65 cm. The experimental procedure was the same for all the experiments, with spatial positions used as stimuli for Experiments 1–3 and orientations used for Experiment S1. On each trial of Experiments 1–3 (Fig 2A), following a 1-s fixation cross, a 40-cm white horizontal axis appeared in the middle of the screen. In the subsequent 25 seconds, 70 red vertical lines were sequentially presented on the axis at different horizontal positions, each for 0.18-s and separated by 0.18-s intervals. The tasks were to report the Mean and Mode of the observed horizontal distribution of the red lines: Subjects first saw a blue vertical line and were required to move it along the axis to locate the Mean (the average horizontal position of the red lines); after completing the estimation of the Mean, they saw a blue box and were instructed to locate it at the position where it would catch the largest number of red lines. To help subjects understand the task, graphed illustrations of mean and mode were given during the instructions. The initial position of the blue line or box was randomly chosen. Subjects used the mouse cursor to move it and left clicked to confirm. No time limit was imposed on either task.

The horizontal coordinates of the 70 red lines on each trial were sampled from a specific underlying distribution, which was a linear combination of multiple beta-like distributions (Fig 2B–2D). Experiments 1–3 differed in the number of components that consisted of the beta-mix distributions: three components for Experiment 1 (“3-beta mix”), four components for Experiment 2 (“4-beta mix”), and a combination of 3-beta and 4-beta mix for Experiment 3.

Each beta-mix distribution had two sets of parameters: shape and weight. The shape parameters, (α, β), controlled the shape of individual beta components and were the same for all the components in the same distribution. The (α, β) could be (3.1, 1.1), (2.9, 2.9) or (1.1, 3.1), respectively corresponding to negatively-skewed, symmetric, and positively-skewed local components of equal variance. In contrast, the weight parameters, (*φ*_1_,*φ*_2_,…,*φ*_*m*_), with $\sum_{i=1}^{m}\varphi_{i}=1$, referred to the relative weight of each component in the mixture distribution, ordered from left to right. The different beta components of a beta-mix distribution had equal widths and joined each other’s ends, whose standard deviations were 0.19 times of their widths.

In Experiment 1, where the distributions were 3-beta mix, the relative weights could be (0.2, 0.3, 0.5), (0.3, 0.2, 0.5), (0.2, 0.5, 0.3), (0.3, 0.5, 0.2), (0.5, 0.2, 0.3), or (0.5, 0.3, 0.2), that is, the full permutation of (0.2, 0.3, 0.5). Each combination of the 3 shape and 6 weight conditions was repeated for 9 times, resulting in 3×6×9 = 162 trials. In Experiment 2, the relative weights for the 4-beta mix were the full permutation of (0.1, 0.2, 0.3, 0.4). Each combination of the 3 shape and 24 weight conditions was repeated twice, resulting in 3×24×2 = 144 trials. Experiment 3 was a combination of 72 trials of 3-beta mix from Experiment 1 (3 shapes × 6 weights × 4 repetitions) and 72 trials of 4-beta mix from Experiment 2 (3 shapes × 24 weights × 1 repetition). The 144 trials were divided into 12 mini-blocks of 10–14 trials, with each mini-block devoted to either 3-beta or 4-beta mix and the two types of mini-blocks interleaved. The existence of mini-blocks was unbeknown to the subject.

The 70 samples for each trial (i.e. the horizontal coordinates of the red lines) were first generated by random and independent draws from its underlying beta-mix distribution, and subsequently scaled to a specific standard deviation and jittered around the center of the screen. The standard deviations of the samples for Experiments 1, 2, and 3 were respectively set to be 7.27, 7.20 and 7.55 cm. Given the screen center as the origin, the samples were allowed to range from –20 to 20 cm and the mean of the samples was within the range of –3.8 to 3.8 cm. In a specific experiment, the same set of samples was used for all subjects, but the order of samples within each trial and the order of the trials were randomized for each subject.

Experiment S1 was a conceptual replication of Experiment 1 where orientations instead of spatial positions were used as stimuli (S8 Fig). Samples were lines of 8 cm long, starting from the center of the screen and pointing to various directions. For a specific subject, all sample lines pointed towards either the upper or lower half of the screen so that the whole range of the stimuli was within 180 degrees. Subjects rotated a line or bar around the origin to report the Mean or Mode of the orientations, analogous to the responding procedures in Experiments 1–3. Half of the subjects reported the Mean first and half reported the Mode first. We did not find any significant differences between these two task orders in subjects’ error patterns, no matter for Mode or Mean estimates.

There were three practice trials preceding the formal experiment. Each experiment took approximately 1.5 hours.

### Data analysis and modeling

#### True mode, mean and skewness

The true mode, mean, and skewness of the distribution were defined based on the 70 samples subjects actually saw, which might be different from those of the underlying distribution. The true mean and skewness were simply the corresponding statistics of the samples. To compute the true mode, we applied the diffusion algorithm of kernel density estimation [89]—a smoothing method performing well for multi-modal distributions—to the samples, with bandwidth parameters chosen automatically by the algorithm. The true mode was defined as the highest peak of the resulting density curve.

#### Clusters-of-samples model for mode estimates

According to the clusters-of-samples (CoS) representation, on each trial, subjects would classify the 70 samples into clusters and maintain the centroids and relative weights of the clusters—${\left\{\left(c_{k},w_{k}\right)\right\}}_{k=1}^{K}$—as the representation for the empirical distribution. In particular, we implemented the clustering process as a distance-dependent Chinese Restaurant Process (ddCRP, [43]) where the number of clusters is controlled by the random process and samples that are close to each other are more likely to be assigned to the same cluster. It should be noted that we used the ddCRP not as a prior for Bayesian inference but as a clustering algorithm.

In the original ddCRP, each sample is attracted by all other samples and may join one of them, otherwise staying alone. Given the large number of samples in our case, for simplicity, we assumed that the probability for a sample to stay alone approaches zero. The probability for the *j*-th sample to join the *l*-th sample (*j*≠*l*) is a function of their distance *d*_*jl*_:

$$P_{join}\left(j,l\right)\propto \exp\left(-d_{jl}^{2}/\gamma \right),$$ (1)

where *γ* is a scaling parameter controlling how quickly the joining probability would decay with distance, and $\sum_{l\neq j}P_{join}\left(j,l\right)=1$. Each sample goes to the same cluster as the sample it joins.

For a specific cluster *k* following the ddCRP, denote the centroid (i.e. mean value) of its samples by *c*_*k*_ and its relative weight (i.e. number of its samples divided by the total number of samples) by *w*_*k*_, where *k* = 1,2,…,*K*. In the CoS model for Mode estimates, we assumed that subjects would report the centroid of the cluster with the highest relative weight, denoted C*.

The ddCRP is a stochastic clustering process, which may lead to different sets of ${\left\{\left(c_{k},w_{k}\right)\right\}}_{k=1}^{K}$ and thus different C* in different runs. In other words, C* is a random variable whose distribution is determined by the empirical distribution of the samples and the distance-scaling parameter *γ*. The distribution of C* has no closed forms and was estimated through Monte Carlo methods: For each specific trial and choice of *γ*, we simulated the clustering process for 1000 times, obtained 1000 sets of ${\left\{\left(c_{k},w_{k}\right)\right\}}_{k=1}^{K}$ and computed the value of C* for each set.

The response in a magnitude estimation task often has a tendency of regression to mean [90]. In all the models, we assume that subjects’ final estimate *Y* undergoes a linear transformation and is contaminated by a Gaussian noise. That is, for the CoS model for Mode estimates,

$$Y=\beta_{0}+\beta_{1}C^{*}+N\left(0,\sigma_{\mathrm{mode}}^{2}\right),$$ (2)

where *β*_0_, *β*_1_, and *σ*_mode_ are free parameters.

In sum, the CoS model for Mode estimates has four free parameters: *γ*, *β*_0_, *β*_1_, *σ*_mode_.

#### Clusters-of-samples model for Mean estimates

In the CoS model for Mean estimates, we assume that subjects would report a weighted average of the centroids of all the clusters. Similar to that of Mode estimates, the modeling of Mean estimates needs to take into account the randomness in clustering.

As elaborated in the Results, we assume that subjects used the same CoS representation—a specific set ${\left\{\left(c_{k},w_{k}\right)\right\}}_{k=1}^{K}$ —for both Mean and Mode estimates. When fitting the CoS model for Mode estimate, we had run the stochastic clustering process for 1000 times and obtained 1000 sets of ${\left\{\left(c_{k},w_{k}\right)\right\}}_{k=1}^{K}$ for each trial and each *γ*. For the best-fit *γ*, we could compute the likelihood that the observed Mode estimate, *y*_mode_, resulted from the *j*-th ${\left\{\left(c_{k},w_{k}\right)\right\}}_{k=1}^{K}$:

$$L_{set}\left(j\right)=\frac{1}{\sqrt{2\pi }\sigma_{\mathrm{mode}}}\exp\left(-\frac{{\left(y_{\mathrm{mode}}-c_{j}^{*}\right)}^{2}}{2\sigma_{\mathrm{mode}}^{2}}\right)$$ (3)

where $c_{j}^{*}$ denotes the centroid of the cluster of the highest weight for the *j*-th ${\left\{\left(c_{k},w_{k}\right)\right\}}_{k=1}^{K}$. That is, given the *y*_mode_ on a specific trial, we could compute the posterior probability of the *j*-th ${\left\{\left(c_{k},w_{k}\right)\right\}}_{k=1}^{K}$ being the CoS representation in use:

$$P_{set}\left(j\right)\propto L_{set}\left(j\right),$$ (4)

with the normalization $\sum_{j=1}^{1000}P_{set}\left(j\right)=1$.

Mode is a local feature of a probability distribution, whose value is determined only by the largest cluster, while mean is a global feature that involves the integration of multiple clusters. We assume that such integration would introduce two additional transformations on the relative weight *w*_*k*_. One transformation maps *w*_*k*_ to $w_{k}^{\alpha }$, which is partly motivated by Stevens’ power law [91]. The power transformation would lead to a subjective weighting of probability that is mathematically equivalent to the form of linear in log-odds [69] widely observed in human judgment and decision-making [65]. Depending on the exponent *α*, small *w*_*k*_ is overweighted and large *w*_*k*_ underweighted, or the reverse. A second transformation on *w*_*k*_ works analogous to the lateral inhibition in perception [92, 93]: The subjective weight of any cluster is reduced by the existence of any other clusters; the closer or the larger the weight of the other cluster, the larger the influence. We assume that the lateral inhibition influences the subjective weight in the form of a shunting inhibition [66], with the inhibitory forces between two clusters decreasing as a Gaussian function of their distance [67]. Combining the two transformations, the subjective weight of *w*_*k*_ has the form:

$$\theta_{k}=A\frac{w_{k}^{\alpha }}{\sum_{j=1}^{K}w_{j}\exp\left(-\frac{{\left(c_{j}-c_{i}\right)}^{2}}{2\sigma_{LI}^{2}}\right)},$$ (5)

where *α* and *σ*_*LI*_ are free parameters, and *A* is a constant to normalize $\sum_{k=1}^{K}\theta_{k}=1$.

For a specific ${\left\{\left(c_{k},w_{k}\right)\right\}}_{k=1}^{K}$ representation, the Mean estimate is thus

$$M^{*}=\sum_{k=1}^{K}c_{k}\theta_{k}.$$ (6)

The final report of the mean is a random mixture of different M* from different ${\left\{\left(c_{k},w_{k}\right)\right\}}_{k=1}^{K}$, weighted by their posterior probabilities (Eq 4), linearly transformed and contaminated by Gaussian noise. The CoS model for Mean estimates has five free parameters: *α*, *σ*_*LI*_, *β*_0_, *β*_1_, *σ*_mean_. See S1 Table for the estimated parameters of the CoS models.

In our modeling, we first used subjects’ Mode estimates to infer their CoS representations and then applied this information to model their Mean estimates. We chose not to do the other way around because mean is a statistic that integrates over multiple clusters and thus provides less diagnostic information for individual clusters than mode does (global versus local). The additional transformations applied to the CoS model for Mean estimates also increases the computational intractability of using Mean estimates to infer subjects’ CoS representations.

#### Subjective weighting model for Mean estimates

The subjective weighting model for Mean estimates assumes that subjects would report the weighted average of all the samples (*N* = 70), with the relative weight of each sample undergoing similar power and lateral inhibition transformations as those of the CoS model. Given that the original weight of each sample is equal (i.e. *ϕ*_*i*_ = 1/*N*, *i* = 1,2,…,*N*), the subjective weight has a similar form as Eq 5 except that the exponent *α* is naturally dropped:

$$\theta_{i}=A\frac{\phi_{i}^{\alpha }}{\sum_{j=1}^{N}\phi_{j}\exp\left(-\frac{{\left(x_{j}-x_{i}\right)}^{2}}{2\sigma_{LI}^{2}}\right)}=B\frac{1}{\sum_{j=1}^{N}\exp\left(-\frac{{\left(x_{j}-x_{i}\right)}^{2}}{2\sigma_{LI}^{2}}\right)},$$ (7)

where *x*_*i*_ denotes the location of the *i*-th sample, *σ*_*LI*_ is a free parameter, and B is a constant to normalize $\sum_{i=1}^{N}\theta_{i}=1$. The final estimate is $\sum_{i=1}^{N}x_{i}\theta_{i}$, linearly transformed and contaminated by Gaussian noise. In sum, the model has four free parameters: *σ*_*LI*_, *β*_0_, *β*_1_, *σ*_mean_.

#### Ideal observer models for mode and mean estimates

In the ideal observer models, we applied the Dirichlet Process Mixture Model (DPMM) to estimate the generative distribution underlying a set of samples. Denote the value of the *i*-th sample by *x*_*i*_, *i* = 1,2,…,*N*. Similar to Orhan and Jacobs [58], the ideal observer’s internal model of the generative process is specified by the following equations:

$$G∼DP\left(G_{0},\alpha \right),$$ (8)

$$G_{0}\left(\mu_{i}\right)=U\left(\mu_{i};a,b\right),$$ (9)

$$\mu_{i}|G∼G,$$ (10)

$$\tau ∼G\left(\tau ;\alpha_{\tau },\beta_{\tau }\right),$$ (11)

$$x_{i}|\mu_{i},\tau ∼N\left(x_{i};\mu_{i},\tau \right).$$ (12)

Here *G* is a distribution of clusters that is itself distributed following a Dirichlet process (DP) with base distribution *G*_0_ and concentration parameter *α*. *μ*_*i*_ denotes the mean of the cluster to which the *i*-th sample belongs. The base distribution *G*_0_(*μ*_*i*_) is a uniform distribution over the interval [*a*,*b*]. The precision *τ* is identical for all clusters and distributed according to a gamma distribution with scale parameter *α*_*τ*_ and shape parameter *β*_*τ*_. The value of the *i*-th sample *x*_*i*_ is generated from a Gaussian distribution with mean *μ*_*i*_ and precision *τ*. The values or priors for the parameters in the generative model had similar settings as those of Orhan and Jacobs[58]: The interval [*a*,*b*] was set to be sufficiently large to include the minimum and maximum sample values in each experiment. *α*_*τ*_ = 1 and a gamma G(1,1) prior was put on *β*_*τ*_. A $G\left(\alpha_{c},1\right)$ prior was put on the Dirichlet process concentration parameter *α*, where *α*_*c*_ was treated as a free parameter.

On each trial, the ideal observer would use the observed ${\left\{x_{i}\right\}}_{i=1}^{N}$ to infer the latent variables of the generative model and thus the Gaussian mixture distribution that has generated the observed samples. The mean and mode of the Gaussian mixture distribution can then be calculated. The Bayesian inference was performed using a Markov chain Monte Carlo (MCMC) algorithm of Neal [94], implemented by Matlab codes adapted from Orhan and Jacobs [58]. For each trial, we obtained 1000 samples from four MCMC chains of 500 samples after burning-in the first 250 samples of each chain.

Following Orhan and Jacobs [58] and others (e.g. [95]), we assume that the ideal observer would average over all posterior samples of the mean and mode as their Mean and Mode estimates. Parallel to the CoS models, we used subjects’ Mode estimates to infer the Gaussian mixture distributions and applied the results to fit subjects’ Mean estimates. In sum, the ideal observer model for Mode estimates has four free parameters: *α*_*c*_, *β*_0_, *β*_1_, *σ*_mode_. The ideal observer model for Mean estimates has three free parameters: *β*_0_, *β*_1_, *σ*_mean_.

#### Other models for mode and mean estimates

The ground-truth and all the moment-based models we considered for Mode and Mean estimates have the general form:

$$Y=\beta_{0}+\sum_{j=1}^{q}\beta_{j}Z_{j}+N\left(0,\sigma^{2}\right),$$ (13)

where *Y* denotes the response (Mode or Mean estimate), *Z*_*j*_ denotes the *j*-th predictor among a total of *q* different predictors that may include the true mode, mean, and skewness of the empirical distribution, *β*_0_, *β*_*j*_, and *σ* are free parameters. The predictors of each model, as suggested by the model name, are described in the Results.

#### Model fitting and comparison procedures

All the models were fitted on the individual level using the maximum likelihood estimate. We used *fminsearchbnd* (J. D’Errico)—a function that implements the Nelder-Mead method and extends the standard Matlab function *fminsearch* to bounded parameters—to search for the parameters that minimized negative log likelihood. To verify that we had found the global minimum, we repeated the search process with different starting points. For the distance-scaling parameter *γ* in the CoS model of Mode estimates, whose change would introduce stochasticity that the Nelder-Mead method cannot handle, we grid searched *γ* ranging from 0.02 to 2.00, optimized the other parameters of the model for each value of *γ*, and chose the combination of *γ* and other parameters that minimized negative log likelihood. We used a similar grid search procedure for the concentration parameter *α*_*c*_ (ranging from 0.10 to 3.00) in the ideal observer model.

For model comparison, we applied AICc—the Akaike information criterion with a correction for finite sample size [61, 62]—to each subject and model as the information criterion for goodness-of-fit. We further calculated the protected exceedance probability based on the group-level Bayesian model selection method [63, 64], which is an omnibus measure across subjects to indicate the probability that a specific model is the best model in the comparison set.

## Supporting information

S1 Table Estimated parameters of the CoS models for mode and mean estimates. (PDF)

S1 Fig Results of Experiment 2 (4-beta mix). (A, C) Subjects’ errors in Mode (A) and Mean (C) estimates varied with the weight condition (abscissa) and the shape condition (different colors). Different weight conditions are arranged from left to right in increasing global skewness. Shaded areas denote 1 SE. (B) The main effect of the shape condition (local skewness) on subjects’ errors in Mode estimates. ns: non-significant, *: *p* < 0.05, **: *p* < 0.01, ***: *p* < 0.001, for Bonferroni corrected post-hoc comparisons. Error bars denote 1 SE.
(D-E) Model comparison results: summed ΔAICc (left, the lower the better) and protected exceedance probability (right, the higher the better). The black bar refers to the protected exceedance probability for the CoS model, and the red bar on the top of the black bar refers to the sum of protected exceedance probabilities of all other models (which is invisible in the Mode plot). For both Mode (D) and Mean (E) estimates, the CoS models fit best to data. GT: ground-truth model; IO: ideal observer model; MoMe: mode+mean model; MeSk: mean+skewness model; MoSk: mode+skewness model; MoMeSk: mode+mean+skewness model; CoS: clusters-of-samples model; SW: subjective weighting model.
(F) Relative frequency of different cluster sizes (number of samples per cluster) estimated for subjects’ CoS representations. For each subject, the relative frequency of each cluster size was averaged across trials and possible CoS representations on each trial. The statistics were then averaged across subjects. Error bars denote 1 SE. Overall, the clustering process tended to generate clusters of small sizes. The frequencies for cluster sizes of 7, 14, 21, and 28—which correspond to the four relative weights used in 4-beta mix—were much higher than those of their neighbors, indicating that the clustering process could partly recover the multimodal structure of the empirical distribution.
(PDF)

S2 Fig Results of Experiment 3 (interleaved 3-beta and 4-beta mix). Plotted in the same format as the results of Experiment 2 (S1 Fig).
(A-C) Subjects’ errors in Mode and Mean estimates for 3-beta trials.
(D-F) Subjects’ errors in Mode and Mean estimates for 4-beta trials.
(G, H) Model comparison results for Mode and Mean estimates.
(I) Relative frequency of different cluster sizes estimated for subjects’ CoS representations, separately for 3-beta (gray bars) and 4-beta (white bars) trials.
(PDF)

S3 Fig Results of Experiment S1 (3-beta mix, orientation). Plotted in the same format as the results of Experiment 2 (S1 Fig).
(A-C) Subjects’ errors in Mode and Mean estimates.
(D, E) Model comparison results for Mode and Mean estimates.
(F) Relative frequency of different cluster sizes estimated for subjects’ CoS representations.
(PDF)

S4 Fig Distributions recovered by ideal observers versus the empirical distributions. (A) Examples for 3-beta distributions. (B) Examples for 4-beta distributions. The gray areas in each panel denote the histogram of the samples presented on a single trial. The generative distribution of the trial is described in the title of the panel (see the legend of Fig 2 for the notations). For example, the generative distribution “LocalNeg, 532” (top left panel) consists of three negatively skewed beta components (“LocalNeg”), with the weights of the three components from left to right being 0.5, 0.3 and 0.2 (“532”). The (α, β) parameters of the LocalNeg, LocalSym and LocalPos beta components were respectively (3.1, 1.1), (2.9, 2.9) and (1.1, 3.1). Dashed curves denote the kernel density of samples. Solid curves denote the posterior density estimation of the Bayesian ideal observer modeled by a Dirichlet Process Mixture Model (DPMM, see the main text). Note that the DPMM posterior density closely matches the empirical kernel density, even for beta mixtures that have skewed beta components.
(PDF)

S5 Fig Intuition of lateral inhibition leading to “skewness preference”. Suppose there are three samples (black lines), with the left two samples closer to each other than to the third, thus forming a positively skewed distribution. If we assume lateral inhibition decreases with distance, the left two samples would exert strong inhibitions on each other, while the inhibitions between them and the third sample would be weaker. Therefore, the left two samples would be underweighted, leading to an overestimation of Mean for positively skewed distributions, and vice versa.
(PDF)

S6 Fig Evidence for shared CoS representations across Mode and Mean estimations. Model comparison results for subjects’ Mean estimates between the original CoS model (“CoS”) and a lesioned model (“CoS w/o Prior”) that does not use the representational information inferred from the Mode estimates on the same trial. (A) Experiment 1. (B) Experiment 2. (C) Experiment 3. (D) Experiment S1. The left and right plots are respectively for summed ΔAICc (the lower the better) and protected exceedance probability (the higher the better). If two distinct CoS representations had been used for Mode and Mean estimates, the CoS representation inferred from the Mode estimate would be non-informative for predicting the Mean estimate and the lesioned model would perform equally well as the original model. In all the experiments, however, the CoS model outperformed the CoS w/o Prior model, providing evidence for a shared CoS representation across the two estimation tasks.
(PDF)

S7 Fig **Model lesion analysis for Experiment 2 (A), Experiment 3 (B) and Experiment S1 (C).** Model comparisons between the CoS model for Mean estimates and its lesioned models: summed ΔAICc (left) and protected exceedance probability (right). The lesioned models included models without Lateral Inhibition (“CoS w/o LI”), without Power Transformation (“CoS w/o PT”), without both the components (“CoS w/o LI&PT”), and without clustering (“SW”, i.e. the subjective weighting model presented in S1E, S2H & S3E Figs). In the protected exceedance probability plots of Experiments 2 and 3, the black bar refers to the protected exceedance probability for the CoS model, and the red bar on the top of the black bar refers to the sum of protected exceedance probabilities of all other models (which is almost invisible for Experiment 3). For Experiment S1, “1” refers to the CoS w/o PT model, “2” refers to the CoS w/o LI model, “3” refers to the CoS w/o LI&PT model. In all the experiments, the original CoS model outperformed the lesioned models, implying that power transformation, lateral inhibition and clustering were all necessary for the CoS model to explain subjects’ Mean estimates.
(PDF)

S8 Fig Stimuli of Experiment S1. Experiment S1 was a conceptual replication of Experiment 1 where orientations instead of spatial positions were used as stimuli. Samples were lines of 8 cm long, starting from the center of the screen and pointing to various directions. For a specific subject, all sample lines pointed towards either the upper or lower half of the screen so that the whole range of the orientations was less than 180 degrees. Subjects rotated a line or bar around the origin to report the Mean or Mode of the orientations. Half of the subjects were required to report the Mean estimate first and the other half the Mode estimate first. Otherwise, the procedure and design were the same as those of Experiment 1 (see Fig 2).
(PDF)

S9 Fig Multimodality of subjects’ Mode and Mean estimates in Experiment 1. To investigate whether the joint distribution of subjects’ Mode and Mean estimates (Fig 5C) was multimodal, we adopted the mixture model clustering method with the integrated completed likelihood criterion (ICL). The “Rmixmod” R package implementing the method was used to evaluate the number of clusters in subjects’ Mode and Mean estimates in different weight conditions. Each panel is for one weight condition, corresponding to that of Fig 5C. Each data point denotes one subject’s Mode and Mean estimates on one trial. The clustering results are presented using symbols of different colors and superimposed ellipses. When a maximum of three clusters were allowed, 2–3 clusters were formed for each weight condition, whose positions agreed with the CoS predictions (Fig 5C, see the main text). ICL: the ICL value of the clustering results presented. ICL_1_: the ICL value for one cluster. That ICL < ICL_1_ indicated that subjects’ Mode and Mean estimates were better fit by multiple clusters than by one cluster and were thus multimodally distributed.
(PDF)
