# Supplementary material for: Human representation of multimodal distributions as clusters of samples
Source: PLoS Comput Biol. 2019 May 14;15(5):e1007047. doi: 10.1371/journal.pcbi.1007047 (PMC6534328; doi:10.1371/journal.pcbi.1007047)
Supplement: S1 Table — (PDF) [file pcbi.1007047.s001.pdf]

Supporting Information

S1 Table. Estimated parameters of the CoS models for Mode and Mean estimates.

Exp	CoS model for Mode estimates				CoS model for Mean estimates				
	γ	β_0	β_1	σ_{mode}	α	σ_{LI}	β_0	β_1	σ_{mean}
**1**	0.29	0.10	0.87	1.15	1.44	3.76	0.11	0.91	1.64
	(0.15~0.58)	(-0.16~0.18)	(0.82~0.93)	(1.00~1.35)	(1.13~2.01)	(1.52~10.45)	(-0.26~0.68)	(0.80~1.00)	(1.35~1.86)
**2**	0.12	0.05	0.97	1.14	1.77	4.11	0.11	1.00	1.79
	(0.07~0.19)	(-0.29~0.49)	(0.90~1.00)	(1.00~1.64)	(1.25~2.00)	(3.50~5.16)	(-0.37~0.44)	(0.77~1.10)	(1.48~1.96)
**3**	0.23	0.00	0.88	1.32	1.44	4.02	0.12	0.94	1.93
	(0.11~1.07)	(-0.19~0.48)	(0.84~0.93)	(1.06~1.68)	(1.19~1.86)	(2.37~7.00)	(-0.33~0.55)	(0.77~1.09)	(1.78~2.11)
**S1**	0.15	0.02	0.97	1.39	1.37	2.86	0.13	1.09	2.27
	(0.12~0.25)	(-0.15~0.24)	(0.93~1.01)	(1.28~1.48)	(0.99~1.61)	(2.08~6.28)	(-0.47~0.55)	(0.86~1.25)	(1.93~3.02)

Note. Numbers outside of the parentheses refer to the median value of subjects' estimated parameters. Numbers in the parentheses refer to the 0.25 and 0.75 quantiles. Parameters of Experiment S1 was transformed to the scale of the other three experiments for the convenience of comparisons across experiments.
