# Supplementary material for: Human representation of multimodal distributions as clusters of samples
Source: PLoS Comput Biol. 2019 May 14;15(5):e1007047. doi: 10.1371/journal.pcbi.1007047 (PMC6534328; doi:10.1371/journal.pcbi.1007047)
Supplement: S1 Fig — (A, C) Subjects’ errors in Mode (A) and Mean (C) estimates varied with the weight condition (abscissa) and the shape condition (different colors). Different weight conditions are arranged from left to right in increasing global skewness. Shaded areas denote 1 SE. (B) The main effect of the shape condition (local skewness) on subjects’ errors in Mode estimates. ns: non-significant, *: p < 0.05, **: p < 0.01, ***: p < 0.001, for Bonferroni corrected post-hoc comparisons. Error bars denote 1 SE. (D-E) Model comparison results: summed ΔAICc (left, the lower the better) and protected exceedance probability (right, the higher the better). The black bar refers to the protected exceedance probability for the CoS model, and the red bar on the top of the black bar refers to the sum of protected exceedance probabilities of all other models (which is invisible in the Mode plot). For both Mode (D) and Mean (E) estimates, the CoS models fit best to data. GT: ground-truth model; IO: ideal observer model; MoMe: mode+mean model; MeSk: mean+skewness model; MoSk: mode+skewness model; MoMeSk: mode+mean+skewness model; CoS: clusters-of-samples model; SW: subjective weighting model. (F) Relative frequency of different cluster sizes (number of samples per cluster) estimated for subjects’ CoS representations. For each subject, the relative frequency of each cluster size was averaged across trials and possible CoS representations on each trial. The statistics were then averaged across subjects. Error bars denote 1 SE. Overall, the clustering process tended to generate clusters of small sizes. The frequencies for cluster sizes of 7, 14, 21, and 28—which correspond to the four relative weights used in 4-beta mix—were much higher than those of their neighbors, indicating that the clustering process could partly recover the multimodal structure of the empirical distribution. (PDF) [file pcbi.1007047.s002.pdf]

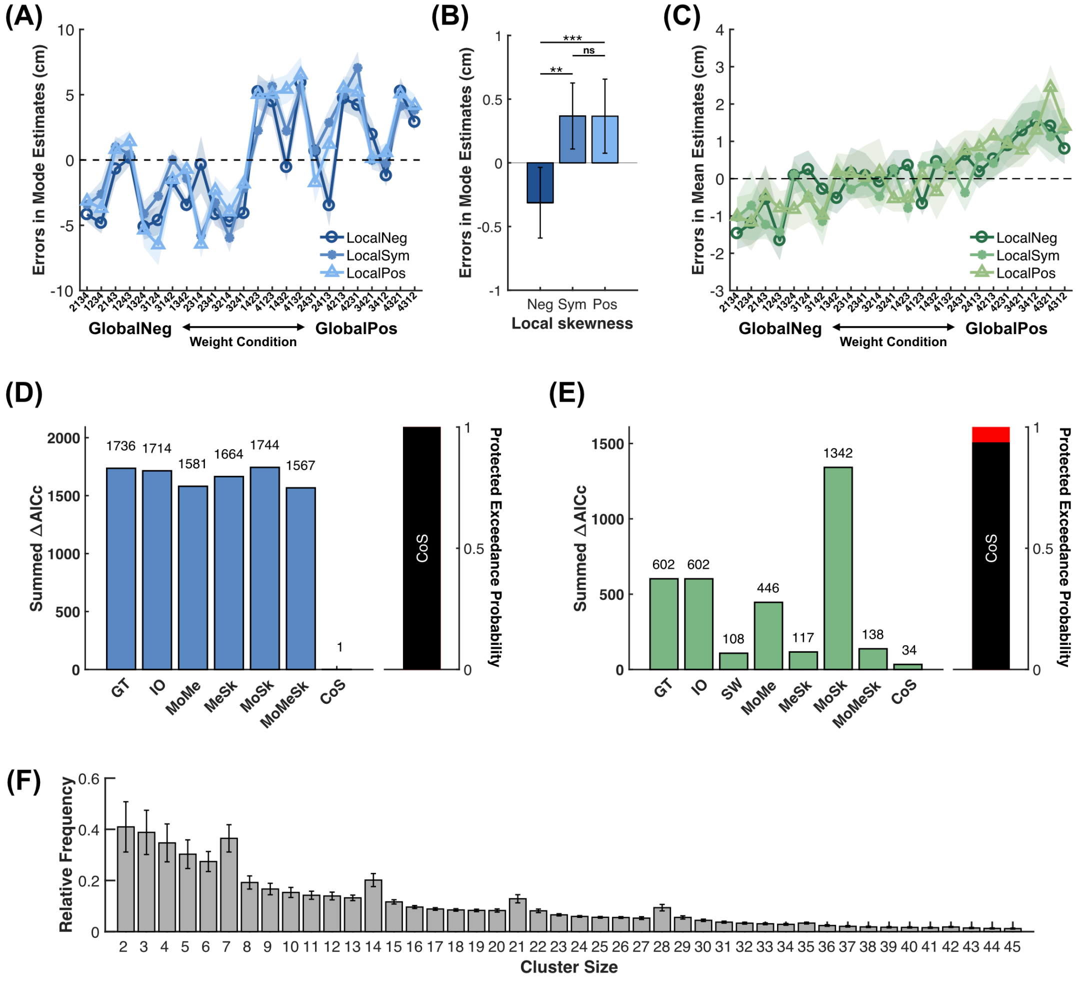

S1 Fig. Results of Experiment 2 (4-beta mix).

(A, C) Subjects' errors in Mode (A) and Mean (C) estimates varied with the weight condition (abscissa) and the shape condition (different colors). Different weight conditions are arranged from left to right in increasing global skewness. Shaded areas denote 1 SE. (B) The main effect of the shape condition (local skewness) on subjects' errors in Mode estimates. ns: non-significant, *: $p < 0.05$, **: $p < 0.01$, ***: $p < 0.001$, for Bonferroni corrected post-hoc comparisons. Error bars denote 1 SE.

(D-E) Model comparison results: summed ΔAICc (left, the lower the better) and protected exceedance probability (right, the higher the better). The black bar refers to the protected exceedance probability for the CoS model, and the red bar on the top of the black bar refers to the sum of protected exceedance probabilities of all other models (which is invisible in the Mode plot). For both Mode (D) and Mean (E) estimates, the CoS models fit best to data. GT: ground-truth model; IO: ideal observer model; MoMe: mode+mean model; MeSk: mean+skewness model;

MoSk: mode+skewness model; MoMeSk: mode+mean+skewness model; CoS: clusters-of-samples model; SW: subjective weighting model.

(F) Relative frequency of different cluster sizes (number of samples per cluster) estimated for subjects' CoS representations. For each subject, the relative frequency of each cluster size was averaged across trials and possible CoS representations on each trial. The statistics were then averaged across subjects. Error bars denote 1 SE. Overall, the clustering process tended to generate clusters of small sizes. The frequencies for cluster sizes of 7, 14, 21, and 28—which correspond to the four relative weights used in 4-beta mix—were much higher than those of their neighbors, indicating that the clustering process could partly recover the multimodal structure of the empirical distribution.
