# Supplementary material for: Human representation of multimodal distributions as clusters of samples
Source: PLoS Comput Biol. 2019 May 14;15(5):e1007047. doi: 10.1371/journal.pcbi.1007047 (PMC6534328; doi:10.1371/journal.pcbi.1007047)
Supplement: S2 Fig — Plotted in the same format as the results of Experiment 2 (S1 Fig). (A-C) Subjects’ errors in Mode and Mean estimates for 3-beta trials. (D-F) Subjects’ errors in Mode and Mean estimates for 4-beta trials. (G, H) Model comparison results for Mode and Mean estimates. (I) Relative frequency of different cluster sizes estimated for subjects’ CoS representations, separately for 3-beta (gray bars) and 4-beta (white bars) trials. (PDF) [file pcbi.1007047.s003.pdf]

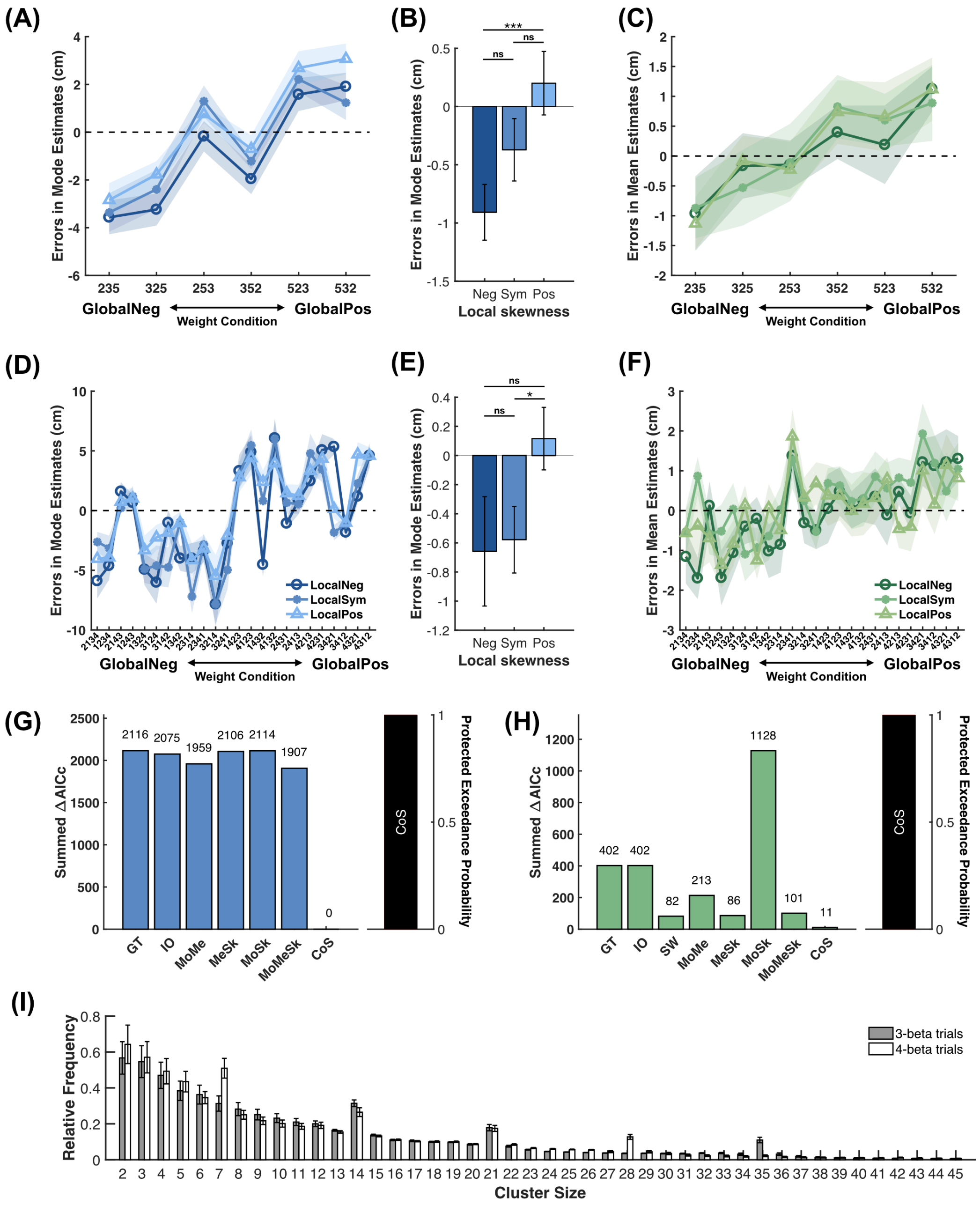

S2 Fig. Results of Experiment 3 (interleaved 3-beta and 4-beta mix).

Plotted in the same format as the results of Experiment 2 (S1 Fig).

(A-C) Subjects' errors in Mode and Mean estimates for 3-beta trials.

(D-F) Subjects' errors in Mode and Mean estimates for 4-beta trials.

(G, H) Model comparison results for Mode and Mean estimates.

(I) Relative frequency of different cluster sizes estimated for subjects' CoS representations, separately for 3-beta (gray bars) and 4-beta (white bars) trials.
