# Supplementary material for: Human representation of multimodal distributions as clusters of samples
Source: PLoS Comput Biol. 2019 May 14;15(5):e1007047. doi: 10.1371/journal.pcbi.1007047 (PMC6534328; doi:10.1371/journal.pcbi.1007047)
Supplement: S3 Fig — Plotted in the same format as the results of Experiment 2 (S1 Fig). (A-C) Subjects’ errors in Mode and Mean estimates. (D, E) Model comparison results for Mode and Mean estimates. (F) Relative frequency of different cluster sizes estimated for subjects’ CoS representations. (PDF) [file pcbi.1007047.s004.pdf]

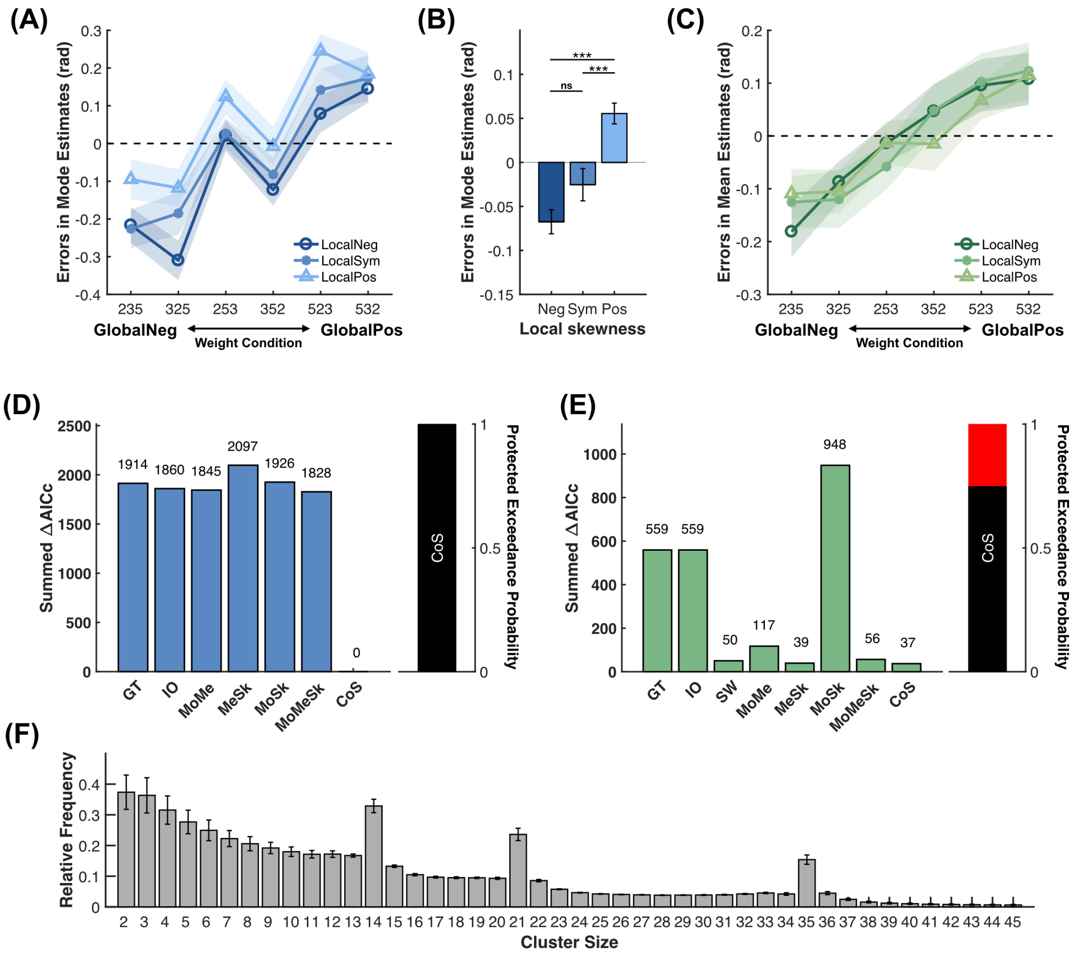

S3 Fig. Results of Experiment S1 (3-beta mix, orientation).

Plotted in the same format as the results of Experiment 2 (S1 Fig).

(A-C) Subjects' errors in Mode and Mean estimates.

(D, E) Model comparison results for Mode and Mean estimates.

(F) Relative frequency of different cluster sizes estimated for subjects' CoS representations.
