# Supplementary material for: Human representation of multimodal distributions as clusters of samples
Source: PLoS Comput Biol. 2019 May 14;15(5):e1007047. doi: 10.1371/journal.pcbi.1007047 (PMC6534328; doi:10.1371/journal.pcbi.1007047)
Supplement: S4 Fig — (A) Examples for 3-beta distributions. (B) Examples for 4-beta distributions. The gray areas in each panel denote the histogram of the samples presented on a single trial. The generative distribution of the trial is described in the title of the panel (see the legend of Fig 2 for the notations). For example, the generative distribution “LocalNeg, 532” (top left panel) consists of three negatively skewed beta components (“LocalNeg”), with the weights of the three components from left to right being 0.5, 0.3 and 0.2 (“532”). The (α, β) parameters of the LocalNeg, LocalSym and LocalPos beta components were respectively (3.1, 1.1), (2.9, 2.9) and (1.1, 3.1). Dashed curves denote the kernel density of samples. Solid curves denote the posterior density estimation of the Bayesian ideal observer modeled by a Dirichlet Process Mixture Model (DPMM, see the main text). Note that the DPMM posterior density closely matches the empirical kernel density, even for beta mixtures that have skewed beta components. (PDF) [file pcbi.1007047.s005.pdf]

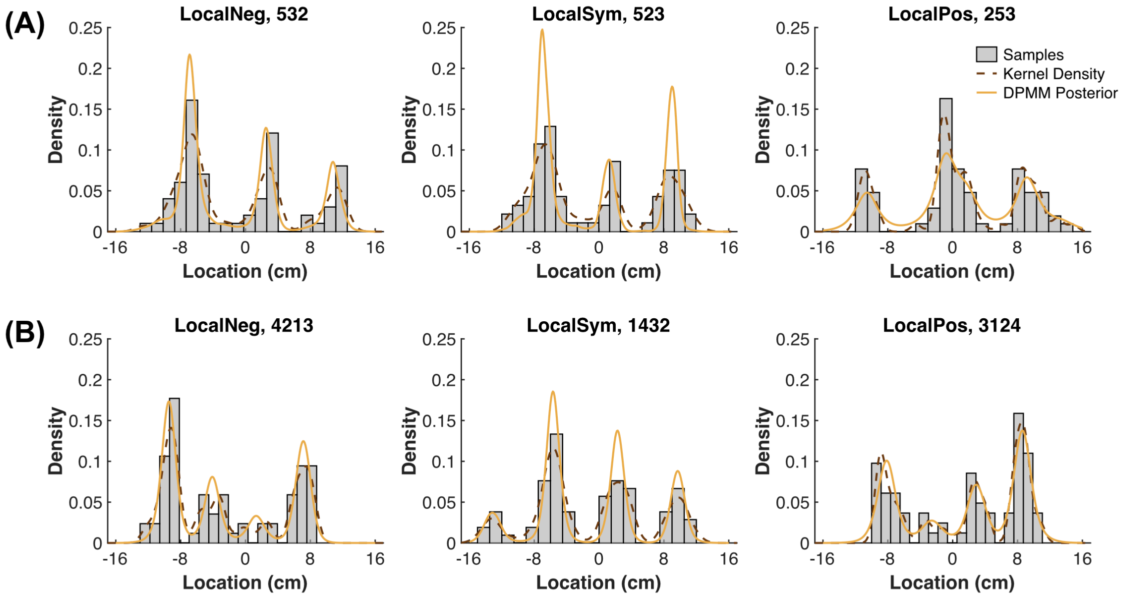

S4 Fig. Distributions recovered by ideal observers versus the empirical distributions. (A) Examples for 3-beta distributions. (B) Examples for 4-beta distributions. The gray areas in each panel denote the histogram of the samples presented on a single trial. The generative distribution of the trial is described in the title of the panel (see the legend of Fig 2 for the notations). For example, the generative distribution "LocalNeg, 532" (top left panel) consists of three negatively skewed beta components ("LocalNeg"), with the weights of the three components from left to right being 0.5, 0.3 and 0.2 ("532"). The (α, β) parameters of the LocalNeg, LocalSym and LocalPos beta components were respectively (3.1, 1.1), (2.9, 2.9) and (1.1, 3.1). Dashed curves denote the kernel density of samples. Solid curves denote the posterior density estimation of the Bayesian ideal observer modeled by a Dirichlet Process Mixture Model (DPMM, see the main text). Note that the DPMM posterior density closely matches the empirical kernel density, even for beta mixtures that have skewed beta components.
