# Supplementary material for: Human representation of multimodal distributions as clusters of samples
Source: PLoS Comput Biol. 2019 May 14;15(5):e1007047. doi: 10.1371/journal.pcbi.1007047 (PMC6534328; doi:10.1371/journal.pcbi.1007047)
Supplement: S5 Fig — Suppose there are three samples (black lines), with the left two samples closer to each other than to the third, thus forming a positively skewed distribution. If we assume lateral inhibition decreases with distance, the left two samples would exert strong inhibitions on each other, while the inhibitions between them and the third sample would be weaker. Therefore, the left two samples would be underweighted, leading to an overestimation of Mean for positively skewed distributions, and vice versa. (PDF) [file pcbi.1007047.s006.pdf]

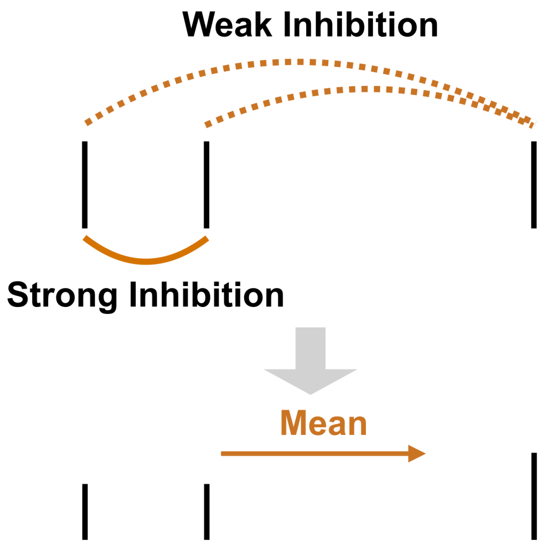

S5 Fig. Intuition of lateral inhibition leading to "skewness preference".

Suppose there are three samples (black lines), with the left two samples closer to each other than to the third, thus forming a positively skewed distribution. If we assume lateral inhibition decreases with distance, the left two samples would exert strong inhibitions on each other, while the inhibitions between them and the third sample would be weaker. Therefore, the left two samples would be underweighted, leading to an overestimation of Mean for positively skewed distributions, and vice versa.
