# Supplementary material for: Human representation of multimodal distributions as clusters of samples
Source: PLoS Comput Biol. 2019 May 14;15(5):e1007047. doi: 10.1371/journal.pcbi.1007047 (PMC6534328; doi:10.1371/journal.pcbi.1007047)
Supplement: S6 Fig — Model comparison results for subjects’ Mean estimates between the original CoS model (“CoS”) and a lesioned model (“CoS w/o Prior”) that does not use the representational information inferred from the Mode estimates on the same trial. (A) Experiment 1. (B) Experiment 2. (C) Experiment 3. (D) Experiment S1. The left and right plots are respectively for summed ΔAICc (the lower the better) and protected exceedance probability (the higher the better). If two distinct CoS representations had been used for Mode and Mean estimates, the CoS representation inferred from the Mode estimate would be non-informative for predicting the Mean estimate and the lesioned model would perform equally well as the original model. In all the experiments, however, the CoS model outperformed the CoS w/o Prior model, providing evidence for a shared CoS representation across the two estimation tasks. (PDF) [file pcbi.1007047.s007.pdf]

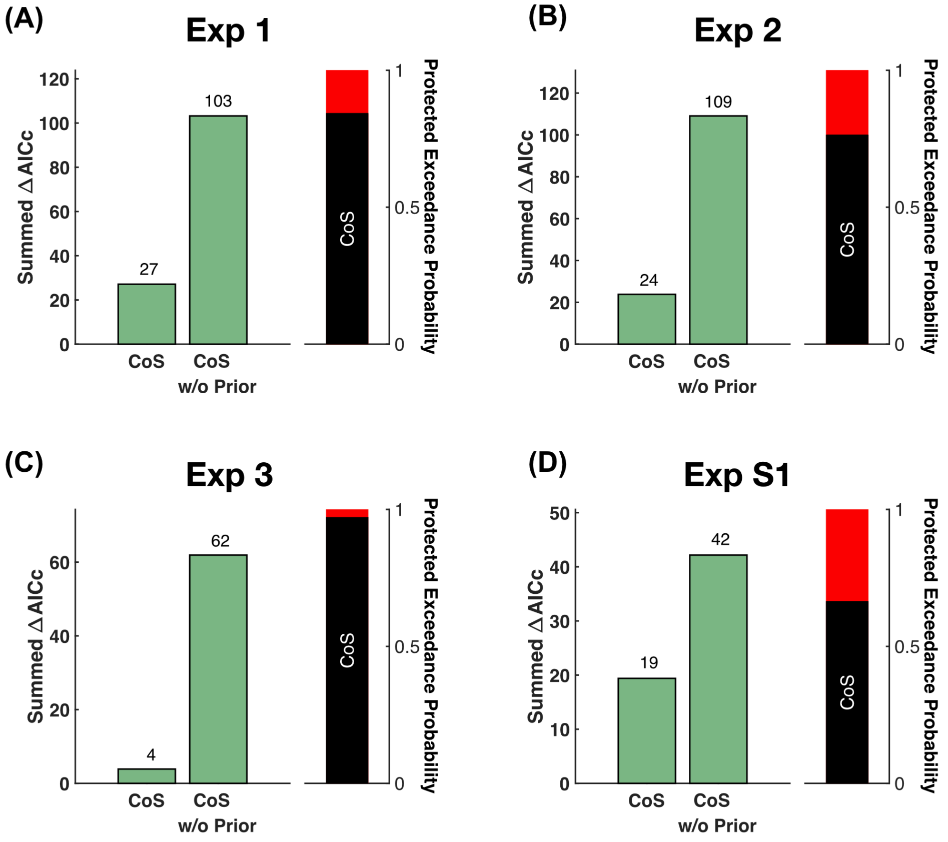

S6 Fig. Evidence for shared CoS representations across Mode and Mean estimations. Model comparison results for subjects' Mean estimates between the original CoS model ("CoS") and a lesioned model ("CoS w/o Prior") that does not use the representational information inferred from the Mode estimates on the same trial. (A) Experiment 1. (B) Experiment 2. (C) Experiment 3. (D) Experiment S1. The left and right plots are respectively for summed ΔAICc (the lower the better) and protected exceedance probability (the higher the better). If two distinct CoS representations had been used for Mode and Mean estimates, the CoS representation inferred from the Mode estimate would be non-informative for predicting the Mean estimate and the lesioned model would perform equally well as the original model. In all the experiments, however, the CoS model outperformed the CoS w/o Prior model, providing evidence for a shared CoS representation across the two estimation tasks.
