# Supplementary material for: Human representation of multimodal distributions as clusters of samples
Source: PLoS Comput Biol. 2019 May 14;15(5):e1007047. doi: 10.1371/journal.pcbi.1007047 (PMC6534328; doi:10.1371/journal.pcbi.1007047)
Supplement: S7 Fig — Model lesion analysis for Experiment 2 (A), Experiment 3 (B) and Experiment S1 (C). Model comparisons between the CoS model for Mean estimates and its lesioned models: summed ΔAICc (left) and protected exceedance probability (right). The lesioned models included models without Lateral Inhibition (“CoS w/o LI”), without Power Transformation (“CoS w/o PT”), without both the components (“CoS w/o LI&PT”), and without clustering (“SW”, i.e. the subjective weighting model presented in S1E, S2H & S3E Figs). In the protected exceedance probability plots of Experiments 2 and 3, the black bar refers to the protected exceedance probability for the CoS model, and the red bar on the top of the black bar refers to the sum of protected exceedance probabilities of all other models (which is almost invisible for Experiment 3). For Experiment S1, “1” refers to the CoS w/o PT model, “2” refers to the CoS w/o LI model, “3” refers to the CoS w/o LI&PT model. In all the experiments, the original CoS model outperformed the lesioned models, implying that power transformation, lateral inhibition and clustering were all necessary for the CoS model to explain subjects’ Mean estimates. (PDF) [file pcbi.1007047.s008.pdf]

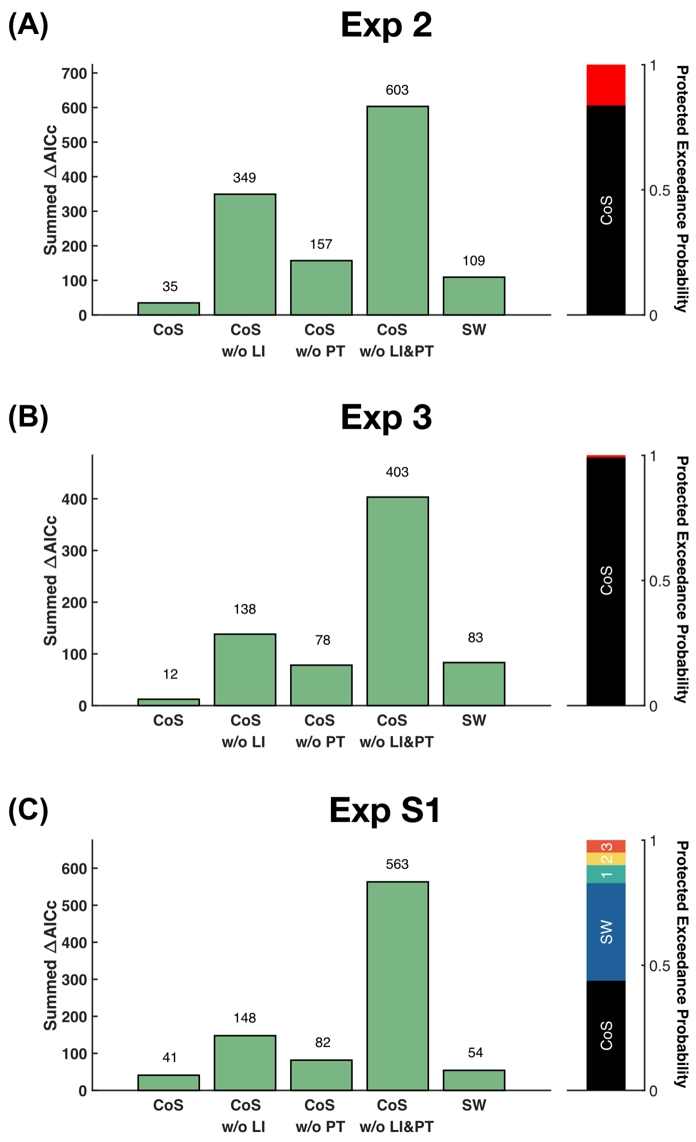

S7 Fig. Model lesion analysis for Experiment 2 (A), Experiment 3 (B) and Experiment S1 (C). Model comparisons between the CoS model for Mean estimates and its lesioned models: summed ΔAICc (left) and protected exceedance probability (right). The lesioned models included models without Lateral Inhibition ("CoS w/o LI"), without Power Transformation ("CoS w/o PT"), without both the components ("CoS w/o LI&PT"), and without clustering ("SW", i.e. the subjective weighting model presented in S1E, S2H, & S3E Figs). In the protected exceedance probability plots of Experiments 2 and 3, the black bar refers to the protected exceedance probability for the CoS model, and the red bar on the top of the black bar refers to the sum of protected exceedance probabilities of all other models (which is almost invisible for Experiment 3). For Experiment S1, "1" refers to the CoS w/o PT model, "2" refers to the CoS w/o LI model, "3" refers to the CoS w/o LI&PT model. In all the experiments, the original CoS model outperformed the lesioned models,

implying that power transformation, lateral inhibition and clustering were all necessary for the CoS model to explain subjects’ Mean estimates.
