# Supplementary material for: Human representation of multimodal distributions as clusters of samples
Source: PLoS Comput Biol. 2019 May 14;15(5):e1007047. doi: 10.1371/journal.pcbi.1007047 (PMC6534328; doi:10.1371/journal.pcbi.1007047)
Supplement: S8 Fig — Experiment S1 was a conceptual replication of Experiment 1 where orientations instead of spatial positions were used as stimuli. Samples were lines of 8 cm long, starting from the center of the screen and pointing to various directions. For a specific subject, all sample lines pointed towards either the upper or lower half of the screen so that the whole range of the orientations was less than 180 degrees. Subjects rotated a line or bar around the origin to report the Mean or Mode of the orientations. Half of the subjects were required to report the Mean estimate first and the other half the Mode estimate first. Otherwise, the procedure and design were the same as those of Experiment 1 (see Fig 2). (PDF) [file pcbi.1007047.s009.pdf]

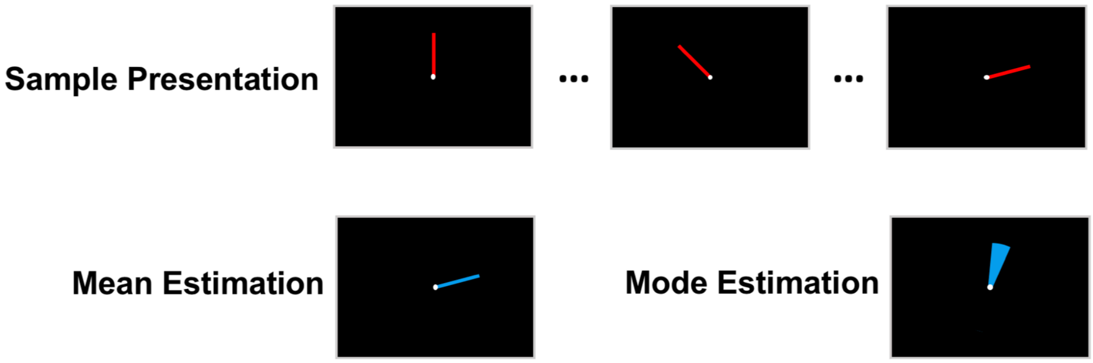

S8 Fig. Stimuli of Experiment S1.

Experiment S1 was a conceptual replication of Experiment 1 where orientations instead of spatial positions were used as stimuli. Samples were lines of 8 cm long, starting from the center of the screen and pointing to various directions. For a specific subject, all sample lines pointed towards either the upper or lower half of the screen so that the whole range of the orientations was less than 180 degrees. Subjects rotated a line or bar around the origin to report the Mean or Mode of the orientations. Half of the subjects were required to report the Mean estimate first and the other half the Mode estimate first. Otherwise, the procedure and design were the same as those of Experiment 1 (see Fig 2).
