# Supplementary material for: Human representation of multimodal distributions as clusters of samples
Source: PLoS Comput Biol. 2019 May 14;15(5):e1007047. doi: 10.1371/journal.pcbi.1007047 (PMC6534328; doi:10.1371/journal.pcbi.1007047)
Supplement: S9 Fig — To investigate whether the joint distribution of subjects’ Mode and Mean estimates (Fig 5C) was multimodal, we adopted the mixture model clustering method with the integrated completed likelihood criterion (ICL). The “Rmixmod” R package implementing the method was used to evaluate the number of clusters in subjects’ Mode and Mean estimates in different weight conditions. Each panel is for one weight condition, corresponding to that of Fig 5C. Each data point denotes one subject’s Mode and Mean estimates on one trial. The clustering results are presented using symbols of different colors and superimposed ellipses. When a maximum of three clusters were allowed, 2–3 clusters were formed for each weight condition, whose positions agreed with the CoS predictions (Fig 5C, see the main text). ICL: the ICL value of the clustering results presented. ICL1: the ICL value for one cluster. That ICL < ICL1 indicated that subjects’ Mode and Mean estimates were better fit by multiple clusters than by one cluster and were thus multimodally distributed. (PDF) [file pcbi.1007047.s010.pdf]

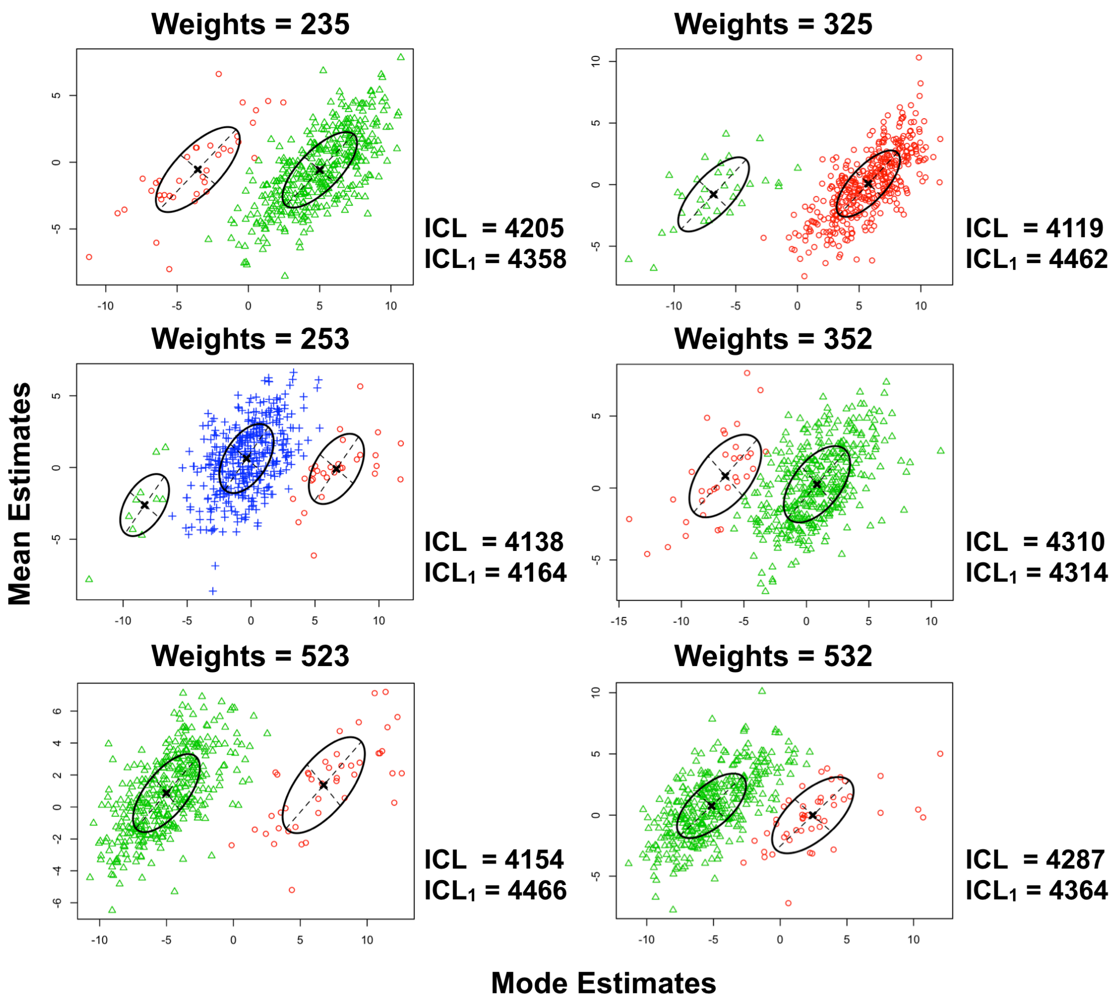

S9 Fig. Multimodality of subjects' Mode and Mean estimates in Experiment 1.

To investigate whether the joint distribution of subjects' Mode and Mean estimates (Fig 5C) was multimodal, we adopted the mixture model clustering method with the integrated completed likelihood criterion (ICL) [1]. The "Rmixmod" R package [2] implementing the method was used to evaluate the number of clusters in subjects' Mode and Mean estimates in different weight conditions. Each panel is for one weight condition, corresponding to that of Fig 5C. Each data point denotes one subject's Mode and Mean estimates on one trial. The clustering results are presented using symbols of different colors and superimposed ellipses. When a maximum of three clusters were allowed, 2–3 clusters were formed for each weight condition, whose positions agreed with the CoS predictions (Fig 5C, see the main text). ICL: the ICL value of the clustering results presented. ICL_1: the ICL value for one cluster. That ICL < ICL_1 indicated that subjects' Mode and Mean estimates were better fit by multiple clusters than by one cluster and were thus multimodally distributed.

References

1. Biernacki C, Celeux G, Govaert G. Assessing a mixture model for clustering with the integrated completed likelihood. IEEE Trans Pattern Anal Mach Intell. 2000;22(7):719-25.
2. Biernacki C, Celeux G, Govaert G, Langrognet F. Model-based cluster and discriminant analysis with the MIXMOD software. Comput Stat Data Anal. 2006;51(2):587-600.
